# Acute and Chronic Effects of Blood Flow Restricted High-Intensity Interval Training: A Systematic Review

**DOI:** 10.1186/s40798-022-00506-y

**Published:** 2022-09-30

**Authors:** Man Tong Chua, Alexiaa Sim, Stephen Francis Burns

**Affiliations:** Physical Education and Sports Science, National Institute of Education, Nanyang Technological University, 1 Nanyang Walk, Singapore, 637636 Singapore

**Keywords:** Blood flow restriction, Occlusion, Interval training, Aerobic exercise, Sprint, Endurance

## Abstract

**Background:**

The implementation of blood flow restriction (BFR) during exercise is becoming an increasingly useful adjunct method in both athletic and rehabilitative settings. Advantages in pairing BFR with training can be observed in two scenarios: (1) training at lower absolute intensities (e.g. walking) elicits adaptations akin to high-intensity sessions (e.g. running intervals); (2) when performing exercise at moderate to high intensities, higher physiological stimulus may be attained, leading to larger improvements in aerobic, anaerobic, and muscular parameters. The former has been well documented in recent systematic reviews, but consensus on BFR (concomitant or post-exercise) combined with high-intensity interval training (HIIT) protocols is not well established. Therefore, this systematic review evaluates the acute and chronic effects of BFR + HIIT.

**Methods:**

The Preferred Reporting Items for Systematic Reviews and Meta-Analyses (PRISMA) guidelines were used to identify relevant studies. A systematic search on 1 February 2022, was conducted on four key databases: ScienceDirect, PubMed, Scopus and SPORTDiscus. Quality of each individual study was assessed using the Physiotherapy Evidence Database (PEDro) scale. Extraction of data from included studies was conducted using an adapted version of the 'Population, Intervention, Comparison, Outcome' (PICO) framework.

**Results:**

A total of 208 articles were identified, 18 of which met inclusion criteria. Of the 18 BFR + HIIT studies (244 subjects), 1 reported both acute and chronic effects, 5 examined acute responses and 12 investigated chronic effects. Acutely, BFR challenges the metabolic processes (vascular and oxygenation responses) during high-intensity repeated sprint exercise—which accelerates central and peripheral neuromuscular fatigue mechanisms resulting in performance impairments. Analysis of the literature exploring the chronic effects of BFR + HIIT suggests that BFR does provide an additive physiological training stimulus to HIIT protocols, especially for measured aerobic, muscular, and, to some extent, anaerobic parameters.

**Conclusion:**

Presently, it appears that the addition of BFR into HIIT enhances physiological improvements in aerobic, muscular, and, to some extent, anaerobic performance. However due to large variability in permutations of BFR + HIIT methodologies, it is necessary for future research to explore and recommend standardised BFR guidelines for each HIIT exercise type.

## Key Points

1. Acute responses of BFR + sprint-based protocols include the acceleration of fatigue mechanisms associated with repeated sprint exercise protocols, possibly impairing performance. However, the extent of impairment differs between upper and lower limbs due to differences in sensitivity to oxygenation and vascular responses.
2. Implementing BFR into HIIT can enhance chronic performance adaptations in aerobic and muscular parameters, whereas improvements in anaerobic components may only be limited to the inclusion of BFR in submaximal exercise interventions.
3. There is a necessity for future research to explore and recommend standardised BFR guidelines for each HIIT exercise type.

## Introduction

High-intensity interval training (HIIT) is a powerful tool in developing an athlete’s cardiorespiratory and metabolic function (aerobic and anaerobic capabilities) which translates to better physical performance [1]. HIIT involves the repetition of short (~ 4-60 s) to long (~ 1-8 min) bouts of high-intensity exercise interspersed with recovery periods. HIIT approaches vary in nature and can include submaximal effort long interval training (LT), short interval training (ST), maximal effort sprint interval training (SIT), repeated sprint training (RST), and mixed-intensity small-sided games (SSG) [1]. Despite the observed benefits of HIIT, athletes, coaches and sport practitioners are still constantly looking for strategies which can enhance and optimise the adaptive responses to training.

In recent years, the implementation of blood flow restriction (BFR) during common exercise modalities (walking, running, cycling and resistance training) has become an increasingly popular, accessible and useful adjunct method in both athletic and rehabilitative settings [2–6]. BFR training involves exercising with the application of an external constricting device (usually blood pressure cuffs or elastic wraps) on the proximal limb musculature, i.e. on the upper arms and/or legs, to restrict arterial blood flow and occlude venous return [7].

The advantages of pairing BFR with aerobic exercise (especially low-intensity aerobic training) have been well documented in recent systematic reviews [3–5]. The advantages are evident in two scenarios: (1) training at lower absolute intensities, for example, walking on gradient with BFR, promotes similar internal training stress, muscular and cardiovascular adaptations akin to that of running-based HIIT [8, 9], and (2) at a similar mechanical or external workload during moderate to high-intensity aerobic training, higher physiological (internal load) stress can be induced with the inclusion of BFR, potentially leading to larger improvements in aerobic, anaerobic and muscular capacities [10–15].

These observations lead into the potential question of whether BFR can be similarly applied during HIIT sessions to further enhance the physiological stimulus and thus adaptive responses of athletes. Recently, literature regarding the use of both BFR + maximal effort sprint training methods like SIT intervals [16] and RST intervals [17], as well as BFR + submaximal effort intervals like LT [11–14, 18], SSG [19, 20] and ST [10], has suggested amplified training benefits in comparison with HIIT without BFR. However, the consensus on the acute mechanisms and chronic effects of these various types of BFR + HIIT protocols (ST, LT, SIT, RST, SSG) are not well-established. Therefore, the main objective of this systematic review was to evaluate the available scientific literature on the acute responses and chronic adaptations to the various BFR + HIIT protocols. Acute responses of BFR + HIIT were analysed according to performance, metabolic (vascular, oxygenation, biochemical and molecular responses), neuromuscular and perceptual variables, while chronic effects of BFR + HIIT were evaluated based on performance (predominantly aerobic, predominantly anaerobic and muscular) adaptations.

## Methods

**Fig. 1:**
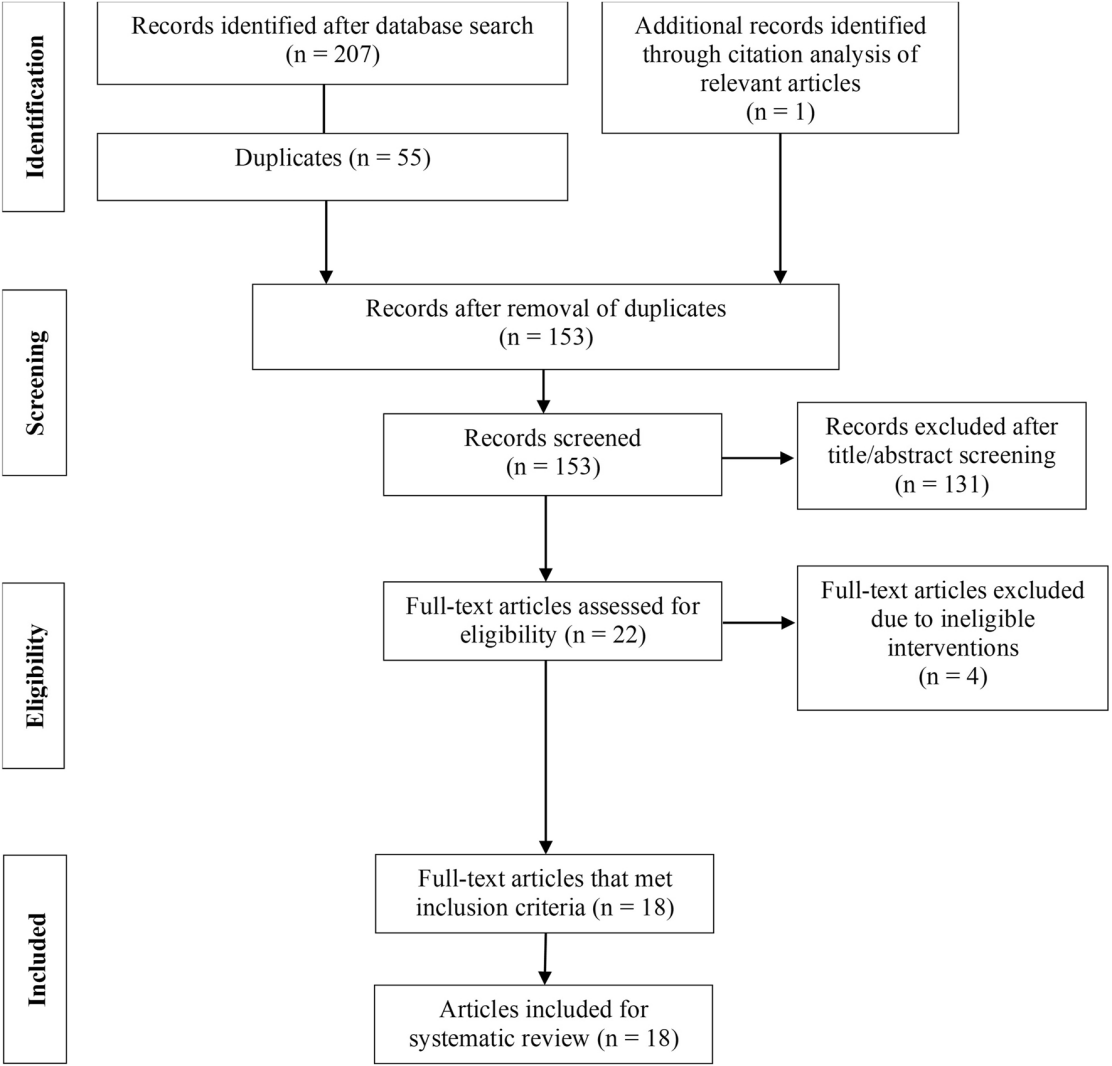
Search strategy and study selection process

This systematic review was conducted in accordance with the Preferred Reporting Items for Systematic Reviews and Meta-Analyses (PRISMA) guidelines [21]. A systematic literature search strategy was performed on 1 February 2022, using a combination of these English descriptors: (occlusion training OR occluded training OR blood flow restricted OR blood flow restriction OR kaatsu) AND (aerobic interval OR games OR interval training OR repeated sprint OR short interval OR long interval OR sprint interval OR run OR cycle OR cycling OR row OR ski) NOT preconditioning. These search terms were agreed on by investigators MC and SB. The search was conducted on ScienceDirect, PubMed, Scopus and SPORTDiscus. The main investigator (MC) conducted the search online independently. All applicable titles and abstracts of the search were uploaded onto Covidence systematic review software (Veritas Health Innovation, Melbourne, Australia) and further screened for relevance by MC and second investigator (AS). Duplicate articles were removed (refer to Fig. 1). The identified articles were then read in entirety, and references of the articles were also reviewed to identify other potentially relevant studies not previously included. The full text review, quality assessment and data extraction were conducted by two independent reviewers (MC and AS), who met to discuss and resolve any discrepancy. If a consensus could not be achieved, discrepancies were resolved with the aid of the final investigator (SB). The inclusion criteria were: (1) original research with human subjects in the age range of 16–50 years; (2) published from 1 January 2000 to 1 February 2022; (3) published in journals indexed in selected databases; (4) evaluated the acute and/or chronic responses effected by BFR interval training; (5) use of practical-BFR (p-BFR), fixed occlusion pressure, pulse elimination pressure (PEP) or arterial occlusion pressure (AOP) methods during exercise; (6) available in English. Articles excluded were: (1) review articles; (2) articles of opinions/viewpoints; (3) validation studies; (4) books or dissertations; (5) case studies; (6) articles that involved the application of BFR in low-moderate intensity interval training (repeated bouts of exercise < 60% $\dot{\mathrm{V}}{\mathrm{O}}_{2\mathrm{max}}$ [22, 23], < 80%$H{\mathrm{R}}_{\mathrm{max}}$ [24]intensity interspersed with rest periods) or any form of continuous training.

### Methodology Quality Assessment: PEDro Scale

Quality assessment of each individual study was completed using the Physiotherapy Evidence Database (PEDro) scale (http://www.pedro.fhs.usyd.edu.au). The PEDro scale objectively assesses experimental studies for their methodological quality—risk of bias, evaluation of internal validity and statistical analysis. It comprises 11 article evaluation items, with 1 point scored for each 'yes' and 0 for each 'no' indicated. As the first item is not calculated in the PEDro score, the minimum and maximum score each article can achieve ranges from 0 to 10, with higher scores indicating greater methodological quality. Scores of < 4 are considered ‘poor’, 4 to 5 are considered ‘fair’, 6 to 8 are considered ‘good’ and 9 to 10 are considered ‘excellent’.

### Data Extraction: PICO Framework

Extraction of data from included studies was conducted using an adapted version of the 'Population, Intervention, Comparison, Outcome' (PICO) framework [25]. For the BFR + HIIT studies, data were extracted and organised as: a) author and year of study, b) participant profile, c) study design (groups), d) BFR methodology (site of BFR, cuff pressure and application procedure), e) exercise intervention (exercise protocol, number of sessions), f) outcome measures, g) significance of outcome measures (*p*-value). Acute responses of BFR + HIIT were evaluated in terms of performance, metabolic (vascular, oxygenation and biochemical and molecular responses), neuromuscular and perceptual variables. Chronic effects of BFR + HIIT were evaluated in terms of performance (predominantly aerobic, predominantly anaerobic and muscular) variables. Findings were classified into the two main categories of (1) acute responses and (2) chronic effects of BFR + HIIT.

## Results

Of the 208 studies identified (Fig. 1), 55 duplicates were removed, and 131 articles excluded after title and abstract screening, leaving 22 studies to be assessed for eligibility. Four studies were excluded due to exercise intervention protocols which did not meet inclusion criteria. Of the eighteen studies evaluated, one reported both acute and chronic effects, five reported acute effects while twelve reported chronic effects of BFR + HIIT..

PEDro scores are evaluated in Table 1 (acute BFR + HIIT studies) and Table 2 (chronic BFR + HIIT studies). In Table 1, all six studies [26–31] scored 6 (‘good’) out of a possible 10. In Table 2, six studies [11–16] scored 5 (‘fair’) while seven studies [10, 17–20, 27, 32] scored 6 (‘good’). All articles did not meet criteria 3 (‘concealed allocation’), 5 (‘blinding of subjects’), 6 (‘blinding of therapists’) and 7 (‘blinding of assessors’). Of the six studies which scored 5, one [15] did not meet criteria 4 (‘groups similar at baseline’) due to a lack of a control (CON) group while one [16] did not meet criteria 2 to randomly allocate the subjects to groups—as participants were highly trained cyclists or triathletes with $\dot{\mathrm{V}}{\mathrm{O}}_{2\mathrm{max}}$ of ≥ 60 $\mathrm{ml}\bullet {\mathrm{min}}^{-1}\bullet {\mathrm{kg}}^{-1}$, they were pair-matched between groups upon initial $\dot{\mathrm{V}}{\mathrm{O}}_{2\mathrm{max}}$, maximal aerobic power (MAP) and critical power (CP). The other four studies [11–14] were derived from a single data collection procedure which had 13 subjects—10 completed (76.9%) and three dropped out. Thus, these studies did not meet criteria 8 which is the 'measure of one key outcome obtained from > 85% initial subjects'.

**Table 1:** Methodological quality of included studies (acute effects of BFR + HIIT) assessed with the PEDro Scale

PEDro*	Taylor et al. [27]	Willis et al. [29]	Peyrard et al. [26]	Valenzuela et al. [28]	Willis et al. [30]	Willis et al. [31]
Eligibility criteria	Yes	Yes	Yes	Yes	Yes	Yes
Randomised allocation	Yes	Yes	Yes	Yes	Yes	Yes
Concealed allocation	No	No	No	No	No	No
Groups similar at baseline	Yes	Yes	Yes	Yes	Yes	Yes
Blind subjects	No	No	No	No	No	No
Blind therapists	No	No	No	No	No	No
Blind assessors	No	No	No	No	No	No
Measure of one key outcome obtained from > 85% initial subjects	Yes	Yes	Yes	Yes	Yes	Yes
Intention-to-treat	Yes	Yes	Yes	Yes	Yes	Yes
Between-group comparisons	Yes	Yes	Yes	Yes	Yes	Yes
Point measures and measures of variability	Yes	Yes	Yes	Yes	Yes	Yes
TOTAL	6	6	6	6	6	6

^*^Eligibility criteria is not calculated in the scores

**Table 2:** Methodological quality of included studies (chronic effects of BFR + HIIT) assessed with the PEDro Scale

PEDro*	Keramidas et al. [18]	Taylor et al. [27]	Behringer, et al. [32]	Paton et al. [10]	Mitchell et al. [16]	Amani-Shalamzari et al. [15]	Amani-Shalamzari, et al. [19, 20]	Christiansen et al. [11–14]	Elgammal et al. [17]
Eligibility criteria	Yes	Yes	Yes	Yes	Yes	Yes	Yes	Yes	Yes
Randomised allocation	Yes	Yes	Yes	Yes	No	Yes	Yes	Yes	Yes
Concealed allocation	No	No	No	No	No	No	No	No	No
Groups similar at baseline	Yes	Yes	Yes	Yes	Yes	No	Yes	Yes	Yes
Blind subjects	No	No	No	No	No	No	No	No	No
Blind therapists	No	No	No	No	No	No	No	No	No
Blind assessors	No	No	No	No	No	No	No	No	No
Measure of one key outcome obtained from > 85% initial subjects	Yes	Yes	Yes	Yes	Yes	Yes	Yes	No	Yes
Intention-to-treat	Yes	Yes	Yes	Yes	Yes	Yes	Yes	Yes	Yes
Between-group comparisons	Yes	Yes	Yes	Yes	Yes	Yes	Yes	Yes	Yes
Point measures and measures of variability	Yes	Yes	Yes	Yes	Yes	Yes	Yes	Yes	Yes
TOTAL	6	6	6	6	5	5	6	5	6

^*^Eligibility criteria is not calculated in the scores

### Acute Responses of BFR-HIIT

#### Methodological Considerations

##### HIIT Exercise Intervention

Six studies (Table 3) reported the acute responses of BFR + HIIT. All six studies [26–31], used maximal, all-out effort, sprint protocols. Five studies adopted RST protocols, four of which utilised repeated sprint tests of 10-s maximal leg- or arm-cycling sprints with 20-s active recovery till volitional exhaustion [26, 29–31], while one used a sport-specific badminton repeated sprint protocol—3 sets of 10 × 10-s all-out sprinting with 20-s rest [28]. Taylor and colleagues [27] employed a SIT protocol of 4 × 30-s maximal cycling sprints, with 4.5-min recovery.

**Table 3:** Acute performance, metabolic, neuromuscular, biochemical, molecular and perceptual responses of BFR + HIIT protocols

References	Participant profile	Study design		BFR methodology				Exercise protocol	Outcomes	*p-*value
				Site of BFR	Cuff Pressure		Application procedure			
Taylor et al. [27]	n=eight healthy trained male cyclists	(1) BFR-SIT (2) SIT (CON)		Proximal portion of each thigh	(1) ~130mmHg (2) No BFR		Inflated within 15-s after each sprint, and 2-min into rest, then deflated after	SIT 4 x 30-s maximal effort cycling sprints, with 4.5min recovery	*Performance response*	
									Total work done and PPO similar between groups (BFR: ~67kJ, ~1147W, CON: ~68kJ, ~1149W) *Molecular responses*	Both *p* > 0.05
									↑* P38MAPK increased in both groups (BFR: 4.1x, CON: 3.2x)	*p *= 0.02
									↑* PGC-1a, VEGF, VEGFR-2 in both groups	All *p *= 0.01
									↑* HIF-1a mRNA at 3h only after BFR	*p *= 0.04
Willis et al. [29]	n=11 healthy, active subjects (six men, five women)	(1) No BFR (CON) (2) 45% BFR (3) 60% BFR		Proximal portion of each thigh	(1) No BFR (2) 45% PEP (3) 60% PEP Exact pressure values not specified		Inflated 5-s before the start of RST to the end of the post-RST measures	RST 10-s all-out maximal cycling sprint with 20-s active recovery to volitional exhaustion	*Performance response*	
									↓* Number of sprints and work done in 45%BFR (~47% and ~53%)) and 60%BFR group (~66% and 69%) as compared to CON	All *p <*0.01
									↓* HR_max_ in 60% BFR compared to CON	*p <* 0.01
									*Perceptual response *	
									↑* RPE legs in both 45% and 60%BFR	Both *p* <0.05
									*Metabolic and oxygenation response*:	
									↓* Peak $\dot{\mathrm{V}}{\mathrm{O}}_{2}$ at 45% BFR (~12.6%) and 60% BFR (~18.2%)	*p* < 0.05
										*p <*0.01
									↓* Δ[HHb] in 60%BFR as compared to CON	*p *<0.001
									↑* Δ[tHb] at 45% and 60%BFR compared with CON	*p *<0.001
									*Neuromuscular responses*:	
									↓*MVC, VAL in both 45% and 60% BFR	Both *p *<0.05
Peyrard et al. [26] ª	n=14 healthy active subjects (ten men, four women)	(1) Normoxia (CON) (2) Normoxia with BFR (N-BFR) (3) Hypoxia (HYP) (4) Hypoxia with BFR (H-BFR)		Proximal portion of the arms	(1) No BFR (2) 45% PEP (95±12mmhg)		Inflated during dynamic warmup, 5-s prior to the neuromuscular testing & first sprint of aRSA test	aRSA 10-s all-out maximal arm-cycling sprint with 20-s active recovery to volitional exhaustion	*Performance responses*	
									↓* Number of sprints in N-BFR (~10) as compared to CON (~13)	*p <* 0.01
									↓* Mean power output of best sprint in N-BFR (~5%) compared to CON	*p =* 0.02
									*Neuromuscular responses:*	
									↑* Δ *Mmax *more impacted with BFR (-9.4%) than CON (+0.8%)	*p* < 0.01
									↑* ΔDb10 from pre to post exercise in occlusion conditions (− 40.8% vs -27.9% without BFR)	*p* < 0.01
Valenzuela et al. [28]ª	n=eight male elite badminton singles players	(1) RS-Normoxia (CON) (2) RS-BFR (3) RS-Hypoxia (RSH)		Proximal portion of upper thighs	(1) No BFR (2) 40% AOP Exact pressure values not specified		Inflated and maintained throughout session, including rest periods	RST (badminton movement) 3 sets, 10 X 10-s all-out sprint, with 20-s rest. 3-min rest between sets	*Performance response*	
									↓* total distance in RS-BFR (1243m) than other groups (CON: 1353m, RSH: 1297m)	Both *p* < 0.05
									↓* mean player load in RS-BFR (1854) compared to CON (2252)	*p* = 0.01
									*Perceptual response *	
									↑* RPE on the legs in RS-BFR (9.5) compared with other groups (both 7)	Both *p *< 0.05
									No differences in overall RPE between RS-BFR (7.6) and CON (8.1)	*p *= .682
									*Biochemical response*	
									↑* bLa in CON than in RS-BFR in the second set	*p* < 0.05
									*Neuromuscular response*	
									↑* jump alteration in RS-BFR (-7.8%) than CON (-2.9%)	*p* < 0.05
Willis et al. [31]ª	n=16 active participants (eleven men, five women)	(1) Normoxia (CON) (2) Normoxia with BFR (N-BFR) (3) Hypoxia (HYP) (4) Hypoxia with BFR (H-BFR)		Proximal portion of the arms	(1) No BFR (2) 45% PEP (93± 12mmhg)		Inflated 5-s before the start of RST and through to the end of the post-RST measures	RST 10-s all-out maximal arm-cycling sprint with 20-s active recovery to volitional exhaustion	*Performance response*	
									Mean power unchanged throughout all conditions	all *p* > 0.05
									Number of sprints similar between CON (12) and N-BFR (9)	all *p* > 0.05
									*Oxygenation responses*:	
									↑* Δ[tHb] during both BFR conditions than without	both *p* < 0.001
									↓* ΔTSI with both BFR conditions than without	both *p* < 0.001
Willis et al. [30]ª	n=seven healthy, active participants (five men, two women)	(1) Normoxia (CON) (2) Normoxia with BFR (N-BFR) (3) Hypoxia (HYP) (4) Hypoxia with BFR (H-BFR)		Proximal portion of limbs (arms and legs)	(1) No BFR (2) 45% PEP Exact pressure values not specified		Inflated 5-s before RST, remained inflated continuously until end of post-RST measures.	RST 10-s all-out maximal leg- and arm-cycling sprint with 20-s active recovery to volitional exhaustion	*Performance response*	
									↓* Number of sprints (leg cycling) in N-BFR (14) than CON (~32)	*p* < 0.05
									↓* Total work done (leg cycling) in N-BFR (72kj) than CON (183kj)	*p* < 0.05
									No change in number of sprints and work done during arm-cycling condition	Both *p* > 0.05
									*Metabolic response*	
									↓* VO_2_(leg cycling) in N-BFR (2597ml/kg/min) than CON (2978ml/kg/min)	*p* < 0.05
									*Oxygenation response*	
									↑* Δ[tHb] (arm cycling) in N-BFR than CON	*p* < 0.001

↑*—significant increase, ↓*—significant decrease, Δ—changes, AOP—arterial occlusion pressure, aRSA—arm repeated sprint ability test, bLa, blood lactate, BFR—blood flow restriction, CON—control, Db10—force evoked by 10 Hz doublets, HHb—deoxygenated hemoglobin, HIF-1a mRNA—hypoxia-inducible factor-1 alpha messenger ribonucleic acid, ${\mathrm{HR}}_{\mathrm{max}}$—maximal heart rate, Mmax—maximal m-wave, amplitude of the muscle compound action potential, MVC—maximal voluntary contraction, P38MAPK—p38 mitogen-activated protein kinases, PEP—pulse elimination pressure, PGC1a—peroxisome proliferator-activated receptor gamma coactivator, PPO—peak power output, RPE—rating of perceived exertion, RS—repeated sprint, RST—repeated sprint training, SIT—sprint interval training, tHb—total hemogoblin, TSI—tissue saturation index, VAL—voluntary activation level, VEGF—vascular endothelial growth factor, VEGFR-2—vascular endothelial growth factor-receptor 2,—$\dot{\mathrm{V}}{\mathrm{O}}_{2}$oxygen uptake,

ª Studies which included either hypoxic and/or hypoxic + BFR conditions, but only comparisons between BFR and CON groups were assessed

##### Participants

The total number of participants was 64: 48 (75%) were male and 16 (25%) were female. Number of participants per study ranged from 7 [30] to 16 [31]. Participants’ age range was between 18 and 39 years. Type of population varied from recreationally active adults [26, 29–31] to well-trained cyclists [27] and elite badminton athletes [28].

##### BFR Application

Sites of BFR application were dependent on the exercise, i.e. BFR on the upper thigh for leg cycling [27–30] and BFR on the upper arm for arm cycling [26, 30, 31]. All studies used pneumatic cuffs, and material was either nylon [26, 27, 29, 30] or not stated [28, 31]. Width of cuffs ranged from 11 to 13 cm (lower limb) [27–30] and 3 to 4 cm (upper limb) [26, 31]. Diameter of cuffs ranged from 85 to 124 cm (lower limb) [27, 29, 30] and 70 cm in upper limbs [30, 31]. Methods of cuff pressure applied were different across studies—fixed pressure [27], PEP [26, 29–31] and AOP [28]. All studies adopted continuous BFR application (participants with BFR cuffs inflated throughout exercise) except for Taylor et al. [27], where an intermittent BFR application was employed (the cuffs were only inflated within 15 s after each sprint, remained inflated for 2 min into rest and deflated before the next bout).

#### Performance Response

##### Number of Sprints and Work Done

The total number of sprints done to volitional exhaustion during the repeated sprint ability (RSA) tests seemed to be significantly affected with the inclusion of BFR during leg-cycling exercise. All three studies which included BFR on lower limbs with RS-type exercises (leg-cycling or badminton-specific movement) reported a significant decrease of sprints and/or work done [28–30]. The studies of Willis et al. [29] and Willis et al. [30] exemplified that when 45%PEP BFR was adopted with RS leg-cycling exercise, total number of sprints and work done decreased by ~ 47–56% (~ 17–18 sprints) and ~ 53–61% (~ 95-111kj), respectively, compared with the CON conditions. A further increase in BFR pressure to 60%PEP caused larger impairments in the number of sprints and work done—decrease of ~ 66% (~ 22 sprints) and ~ 69% (~ 120kj) ,respectively, as compared with the CON condition [29]. Similarly, during RST where the number of badminton-specific sprints (3 sets of 10 × 10-s all-out sprints with 20-s rest) were fixed, total distance achieved was significantly lower in the BFR condition (~ 1243 m) than CON condition (~ 1353 m) [28]. For SIT protocol of 4 sets of 30-s maximal cycling (with 4 min 30 s of passive recovery), total work done was similar between the BFR and CON group [27]. It is important to highlight that in this last study, participants in the BFR condition only had cuffs inflated within 15 s after the maximal sprints, and 2 min into rest, which may have possibly preserved performance.

These results from lower body exercise contrast with the inclusion of BFR on upper limbs for RS exercise (arm cycling) to exhaustion. Two out of three studies utilising arm cycling reported that the number of sprints and work done were similar to CON [30, 31], while one study reported a significant decrease in number of sprints performed (~ 23%; BFR: 10 vs CON: 13 sprints) with BFR [26]. The differences between lower and upper body exercise do not appear to be explained by cuff pressure, period of cuff inflation, or types of sprints performed which were similar. Potential reasons for the difference between repeated sprint performance in lower and upper limbs will be discussed in the Metabolic Responses section.

##### Power Output

Peak power output (PPO) during exercise was only measured in one study [27], and was similar between conditions (BFR: ~ 1147 W vs CON: ~ 1149 W). As mentioned above, BFR pressure was gradually applied after the 30-s maximal sprint effort and for only part of the rest period which may have allowed for sufficient recovery of energy systems between exercise bouts. Three studies reported similar mean power output (MPO) throughout RSA (leg- and arm-cycling) tests in both BFR and CON conditions [29–31]. However, in the study of Peyrard et al. [26], when MPO of the best arm-cycling sprint was compared between BFR and CON conditions, it was significantly reduced by occlusion (BFR: ~ 520 W vs CON: ~ 547 W).

#### Metabolic Responses

##### Oxygen Uptake

Three studies measured the peak $\dot{\mathrm{V}}{\mathrm{O}}_{2}$ response (highest 30-s oxygen uptake) during the RSA test in both BFR and CON conditions [29–31]. These studies noted a disparity between peak $\dot{\mathrm{V}}{\mathrm{O}}_{2}$ achieved when BFR was included with a leg-cycling RSA test compared with BFR during an arm-cycling RSA test. Willis and colleagues [29] investigated BFR leg cycling during RSA test at 0%, 45% and 60% PEP and observed that peak $\dot{\mathrm{V}}{\mathrm{O}}_{2}$ in the 45% and 60% BFR condition were ~ 12.6% and ~ 18.2% lower than CON condition. With higher and more severe occlusion pressure, it was observed that participants were unable to exhaust both the cardiovascular and respiratory systems due to possible limitations and fatigue at the peripheral level [29]. Likewise, another study by Willis et al. [30] found a ~ 12.8% decrease in peak $\dot{\mathrm{V}}{\mathrm{O}}_{2}$ compared with CON, when BFR at 45% PEP was included during leg-cycling RST. However, when arm-cycling RST was conducted on the participants, peak $\dot{\mathrm{V}}{\mathrm{O}}_{2}$ was similar in both BFR and CON conditions [30, 31]. The difference between oxygen uptake in BFR + RS arm and leg cycling was hypothesised for several reasons. First, arms display greater sensitivity to oxygenation than legs during maximal exercise due to higher oxygen demand per unit of muscle. Given that relative workload was similar (maximal effort and same exercise duration), the characteristics of the arms, i.e. smaller muscle mass, vessel diameter and lower vascular conductance, would generally lead to lower oxygen extraction and lower perfusion per kg in arms than legs [33, 34]. However, a greater hyperaemic effect and vascular regulation were observed in the skeletal muscle of arms than legs—higher blood volume concentrations, activation of muscle pump, and higher conduit vessel dilation of the brachial arteries of the arm (as compared to femoral arteries in the legs) during dynamic exercise—which maintained the oxygen delivery of the arms more so than the legs [35, 36]. During BFR conditions, the vascular regulation of blood flow in the arms versus legs was accentuated, appearing to be at a higher rate in the arms than legs. The greater vascular resistance (ratio of mean arterial pressure to blood flow) imposed by BFR likely caused an increase in blood volume in the muscle tissue, which altered the perfusion pressure to increase oxygen extraction [30].

##### Oxygenation/Vascular Responses—Pulse, Cerebral, Muscle

Acute oxygenation responses measured at the pulse, cerebral and muscular level during exercise allow scientists to better understand and interpret the physiological mechanisms that underpin adaptations after a training intervention—in this case, BFR + RS exercise.

All five studies that measured pulse oxygen saturation (Sp ${\mathrm{O}}_{2}$) via an oximeter (attached to the finger or earlobe) during RS exercise on either lower and/or upper limbs reported that it was not impacted by the use of BFR when compared to a CON condition [26, 28–31]. Unlike in conditions of systemic hypoxia where a reduction in Sp ${\mathrm{O}}_{2}$ and higher post-exercise blood flow (caused by hypoxia-induced vasodilation) after RS exercise were observed, BFR (localised hypoxia) conditions demonstrated similar Sp ${\mathrm{O}}_{2}$ levels and seemingly lower post-exercise blood flow as compared to CON conditions [31]. Willis et al. [31] suggested that under partial BFR occlusion where blood flow is limited, different haemodynamic and vascular responses are elicited to control the changes of blood flow and alteration in oxygen delivery during RS exercise. A possible response to control the increased changes in blood volume and maintenance of Sp ${\mathrm{O}}_{2}$ during BFR and RST was proposed to be the continual shifts in perfusion pressure gradient, rather than cardiac output and local muscle vasoconstriction, which are both limiting factors of blood flow during high-intensity exercise [37].

Near-infrared spectroscopy (NIRS) was used to measure cerebral and muscular oxygenation. Three studies measured cerebral oxygenation responses during RS exercise [26, 29, 30], one study during both arm and leg cycling [30]. During leg-cycling RS exercise, two studies observed no significant differences between BFR and CON conditions in measurements of changes in concentration for total haemoglobin (ΔtHb), deoxyhaemoglobin (ΔHHb), oxyhaemoglobin (ΔO_2_Hb) and absolute maximum tissue saturation index (TSI) [29, 30]. It was suggested that in leg-cycling RS, changes in cerebral blood volume ΔtHb increase near exhaustion no matter the condition [29] and are likely due to neural-vascular regulatory coupling which causes an increase in cerebral blood flow to maintain oxygen delivery [38]. In the two studies that investigated arm-cycling RST, one observed no differences between BFR and CON conditions for measurements of cerebral ΔHHb, ΔO_2_Hb and TSI [30], and one observed no differences in the changes in tissue saturation index on the pre-frontal cortex (ΔTSIpfc) [26]. These results indicated that the use of BFR did not induce any noteworthy changes in central oxygenation responses [26].

With regard to muscle oxygenation responses, there were mixed results. In badminton-specific RST, muscle oxygen saturation (Sm ${\mathrm{O}}_{2}$) was not different between BFR and CON conditions [28]. This differed from a previous study that reported a lower Sm ${\mathrm{O}}_{2}$ in individuals performing leg extensions with BFR [39]. The authors debated that the lack of Sm ${\mathrm{O}}_{2}$ differences between BFR and CON may be due to the maximal intensity of RS-exercise overshadowing the ''hypoxic' effects of BFR + RST, or the biomechanical nature of badminton RS movements altering Sm ${\mathrm{O}}_{2}$ kinetics [28]. Three studies reported findings in BFR + RS arm cycling [26, 30, 31], while two studies investigated leg-cycling BFR + RS [29, 30]. Both Peyrard et al. [26] and Willis et al. [31] observed that tissue saturation index of the biceps brachii (TSI^bb^) was impacted by BFR (lower muscle oxygenation) at rest (pre-RSA test) as compared to CON. However, there was disagreement between the two studies in the ΔTSI^bb^ after BFR + RS, as compared with CON. Peyrard et al. [26] reported that ΔTSI^bb^ was not impacted by BFR and attributed this observation to the mechanisms of maximal exercise—which induced vasodilation and an increase in arterial pressure sufficient to counteract the action of BFR on tissue oxygenation parameters. However, in the study of Willis et al. [31], a significantly lower ΔTSI^bb^ and greater ΔtHb were observed in BFR compared with CON after RST. It was proposed that BFR may have increased muscle oxygen extraction closer to maximal capacity and perfusion pressure could have been the main mechanism for increased changes in blood volume, rather than cardiac output or local muscle vessel vasoconstriction.

In the study of Willis et al. [30], comparisons of muscle oxygenation for arm- and leg-cycling RSA tests were conducted. Investigators observed a greater ΔtHb in the biceps brachii and vastus lateralis in BFR as compared to CON in arm and leg cycling, respectively. This observation substantiates the idea that BFR conditions accentuate vascular regulation of blood flow due to higher vascular resistance, increasing blood volume, and thus perfusion pressure to increase oxygen extraction. Furthermore, two BFR conditions (45%PEP and 60%PEP) were also compared with CON in leg-cycling RST [29]. Like arm cycling, greater ΔtHb was observed in the vastus lateralis in both 45% and 60%BFR compared with CON.

Absolute maximal TSI values of the vastus lateralis were significantly lower in the 60%BFR than the 45%BFR and CON conditions. A significantly lower ΔHHb of the vastus lateralis and a significantly greater ΔO_2_Hb were observed at 60% BFR compared with CON. Collectively, these observations depict that as severity of BFR increases, a possible increase in oxygen extraction is required to maintain maximal sprint performance.

##### Biochemical Responses

Four studies compared blood lactate (bLa) response during RS exercise in BFR and CON conditions [28–31]. There were no differences between bLa responses between BFR and CON conditions during RS exercise using arm-cycling [30, 31], leg-cycling [29, 30] or badminton-specific movements [28]. Moreover, Valenzuela et al. [28] observed that there were no differences between BFR and CON in creatine kinase (CK) activity, despite increases in both conditions 24- and 48- h after the session.

##### Molecular Responses

In the study of Taylor et al. [27], activation of p38 mitogen-activated protein kinases (p38MAPK) and angiogenic messenger ribonucleic acid (mRNA) expression were investigated post-exercise. These measures were conducted to identify if exercise-induced capillary growth (angiogenesis) would be potentially induced in response to different physiological stresses during intense exercise. Phosphorylation of p38MAPK as well as mRNA expression, peroxisome proliferator-activated receptor γ coactivator-1α (PGC-1α), vascular endothelial growth factors (VEGF) and its receptors (VEGFR-2) significantly increased immediately after both CON and BFR exercise interventions, and returned to baseline at 3 h post-exercise, but there were no differences between the two conditions. Hypoxia-inducible factor-1α (HIF-1α) mRNA expression—known to upregulate several genes to promote adaptations to metabolic stresses imposed by hypoxic conditions—increased at 3 h only after BFR, which may suggest possible stimulus for hypoxia-mediated skeletal muscle remodeling to increase capillary density.

#### Neuromuscular Responses

It is understood that both peripheral and central fatigue contribute to the decrease of maximal voluntary contraction (MVC) force after leg-cycling [40] and arm-cycling sprints [41], Two studies evaluated neuromuscular responses during RST—one on leg cycling [29], and one on arm cycling [26]. Willis et al. [29] reported a significant decrease in MVC and voluntary action level (VAL) in 45%PEP and 60%PEP BFR as compared with CON. Between 45%PEP and 60%PEP, 60%PEP demonstrated a larger decrease in MVC and VAL. The root mean square of muscle compound action potential (RMS/M-wave), the summated action potentials of stimulated motor neurons, in 60%PEP BFR was also significantly lower compared with CON. The ratio of resting stimulations at 10 Hz over stimulation at 100 Hz (P10/P100) significantly decreased across all conditions, aligning with the decrease in leg-cycling RS performance over time due to peripheral fatigue.

As depicted in the heavily impacted sprint performance, the BFR conditions may have also impacted supraspinal fatigue, as demonstrated in the reduction in responses of central parameters (VAL, RMS/M-wave), which could have been caused by inhibitory signals from type III and IV afferents after the large increase in cerebral blood volume [42–44]. In the study by Peyrard et al. [26] using RS arm cycling, the investigators observed no significant differences between BFR and CON conditions in neuromuscular measurements from central indices. However, they did find a difference in two peripheral indices—change in force amplitude for paired electrical muscle stimulation at 10 Hz (ΔDb10) and change in amplitude of muscle compound action potential (Δ ${\mathrm{M}}_{\mathrm{max}}$) were impacted by occlusion to a greater extent from pre- to post-exercise with BFR (ΔDb10: − 40.8 ± 4.7% (BFR) vs. − 27.9 ± 4.5% (CON), ΔM_max_: − 9.4 ± 1.9% (BFR) vs + 0.8 ± 2.0% (CON)). The higher decrease of ΔDb10 indicates that the onset of peripheral fatigue occurred more rapidly in the BFR than CON condition and indicates that this occurred at and/or beyond the sarcolemma, partially due to impairments in muscle excitation–contraction coupling. This is potentially due to increased severity of metabolic processes like intracellular accumulation of hydrogen ions [45], reduction in bLa removal [46] and faster phosphocreatine breakdown [47]. Also, the greater impairment of the amplitude of muscle compound action potential (${\mathrm{M}}_{\mathrm{max}}$) in BFR, in contrast to CON, could be attributed to the imbalance of ion concentrations across the muscle membrane, likely due to a larger increase in sarcolemmal permeability [48], which is imposed by additional muscle damage in BFR conditions [49].

In a badminton RS protocol, there was a significantly greater decrement in jump height from pre to post in a countermovement jump test with BFR as compared to CON conditions [28]. The investigators similarly proposed that BFR RS exercise led to greater fatigue associated with marked accumulation of intramuscular metabolites and greater decrease in muscle pH.

Thus, it appears that BFR during lower-limb RS performance may be heavily impaired by factors affecting both peripheral and central fatigue etiology while for BFR upper-limb RS, the extent of performance impairment is comparatively lower, and more likely by factors influencing peripheral fatigue than central mechanisms. More investigations are required to confirm the fatigue etiology within lower-limb and upper-limb RS exercise.

#### Perceptual Responses

Four studies investigated the ratings of perceived exertion (RPE) between CON and BFR conditions during RS exercise protocols [28–31]. During arm-cycling RST, RPE arms and RPE breathing were not significantly affected by BFR occlusion compared with CON [30, 31]. In leg-cycling RST, results were mixed. One study reported that RPE legs and RPE breathing were not affected by occlusion [30], while another study observed that despite no significant differences in RPE breathing, RPE legs were significantly affected by occlusion at 45% PEP (RPE: 19.5 ± 0.7) and 60% PEP (RPE: 19.5 ± 0.6) as compared to CON (17.7 ± 2.0) [29]. In a badminton RS protocol, RPE legs were reported to be significantly higher in BFR (9.5 ± 0.5) than CON (7.0 ± 1.3), although overall RPE was similar in both conditions [28]. As observed, perceptual responses were not affected during upper-limb BFR-RS exercise and likely to be negatively affected when lower-limb BFR-RS protocols were adopted—which may be due to the differences in metabolic, oxygenation and vascular responses between the upper and lower limbs.

### Chronic Effects of BFR + HIIT

#### Methodological Considerations of Included Studies

##### BFR + HIIT Exercise Intervention

There were 13 studies included (refer to Table 4) which investigated the chronic effects of BFR + HIIT, all of which employed different BFR + HIIT exercise protocols [10–20, 27, 32]. Four studies incorporated BFR into sprint-based exercise protocols like cycling SIT [16, 27], submaximal effort sprint training (running) [32] and basketball-specific RST [17]. Seven studies adopted a combination of BFR and submaximal aerobic intervals—six of which utilised LT in cycling [11–15, 18], and one employed ST in running [10]. Four papers from Christiansen et al. [11–14] were published based on data derived from a single study data collection. The last two studies [19, 20] likewise obtained data collected from the same data collection process, where BFR was included into a futsal SSG protocol.

**Table 4:** Chronic performance (aerobic, anaerobic, muscular) effects of BFR + HIIT protocols

References	Participant profile	Study design	BFR methodology			Exercise intervention		Performance outcomes	*p-value*
			Site of BFR	Cuff pressure	Application procedure	Number of sessions	Exercise protocol		
Keramidas et al. [18]	n = 20 healthy, untrained subjects (6 men, 14 women)	(1) BFR (2) CON	Proximal portion of each thigh	(1) 90 mmHg (2) No BFR	Pneumatic cuffs, inflated during exercise bouts, depressurised during active recovery	18 sessions, 3 sessions per week for 6 weeks	Bouts of 2-min cycling (90% VO _2__max_ OR VO _2__max_PRESS), 2-min recovery (50% VO _2__max_ OR VO _2__max_PRESS)	*Aerobic*	
								No changes in $\dot{\mathrm{V}}{\mathrm{O}}_{2\mathrm{max}}$, HR_max_, RPE, RPE (legs), AT in both groups	All *p* > 0.05
								↑* MAP (CON: 15%; BFR: 25%), VE_max_ in both groups	Both *p* < 0.001
								↓* $\dot{\mathrm{V}}{\mathrm{O}}_{2}$ (from ~ 78% to ~ 72%) during submaximal test in both groups	All *p* < 0.05
								↑* TTF at 150%MAP in both groups	*p* < 0.05
Taylor et al. [27]	n = 20 healthy trained male cyclist	(1) BFR (2) CON	Proximal portion of each thigh	(1) ~ 130 mmHg (2) No BFR	Pneumatic cuffs, inflated within 15 s after each sprint, 2-min into rest	8 sessions, 2 sessions per week for 4 weeks	SIT: 30-s maximal effort sprint cycling, with 4.5 min recovery 4, 5, 6, 7 sets	*Aerobic*	
								↑* MPO during training in CON than BFR	*p* < 0.01
								↑* $\dot{\mathrm{V}}{\mathrm{O}}_{2\mathrm{max}}$ (4.5%) only in BFR	*p* = 0.01
								No changes in 15 km TT performance time	*p* > 0.05
								*Anaerobic*	
								↑* Sprint PPO in both groups (CON: 6.8%; BFR: 6.4%)	*p* = 0.02
Behringer et al. [32]	n = 25 healthy male sport students	(1) BFR (2) CON	Proximal part of upper thighs	(1) Moderate perceived pressure (7/10) (2) No BFR	p-BFR, wrapped during entirety of exercise duration	12 sessions, 2 sessions a week for 6 weeks	6 sets of 100 m sprints at 60–70% best sprinting time, with 1-min recovery	*Anaerobic*	
								↓* Sprint times in BFR (− 0.38 s, 3%) as compared to CON (− 0.16 s, 1.3%)	*p* < 0.05
								*Muscular*	
								↑* RFD in BFR (6kN/s, 24.9%) more than CON (0.4kN/s, 1.7%)	*p* = 0.02
								↑* Muscle thickness of rectus femoris in BFR	*p* = 0.004
Paton et al. [10]	n = 16 healthy, active subjects (10 males, 6 females)	(1) BFR (2) CON	Proximal portion of thighs	(1) Moderate perceived pressure (7/10) (2) No BFR	p-BFR, wrapped during exercise bout, removed between sets	8 sessions, 2 sessions per week for 4 weeks	30-s running at 80% PRV), 30-s rest 2 sets of 5 reps in session 1 to 3 sets of 8 reps in session 8	*Aerobic*	
								↑ $\dot{\mathrm{V}}{\mathrm{O}}_{2\mathrm{max}}$ (BFR:6.4% vs CON:4.0%) and TTE (BFR:26% vs CON: 17%) in both groups	All between group *p* > 0.05
								↑ PRV (BFR:3.6% vs CON:1.4%) incremental run time (BFR:6.1% vs 2.0%) in both groups	
								↑ RE (-6.7%) only in BFR	
Amani-Shalamzari et al. [15]ª	n = 32 healthy active collegiate females	All BFR (1) IP-CE (2) CPP-IE (3) CPC-IE (4) IP-IE	Proximal portion of thighs	Varies on condition Refer to article for exact BFR protocols	Pneumatic cuffs, inflated during exercise bouts, deflated during recovery	12 sessions, 3 sessions per week for 4 weeks	2-min running with 1-min recovery × 10 sets except for IP-IE group Exercise intensities vary depending on group	*Aerobic*	
								↑*$\dot{\mathrm{V}}{\mathrm{O}}_{2\mathrm{max}}$ ( IP-CE: 9.6%; CPP-IE: 11.2%; CPC-IE: 14.8%; IP-IE: 8.4%) and v $\dot{\mathrm{V}}{\mathrm{O}}_{2\mathrm{max}}$ in all groups	All *p* < 0.05
								↑*%RE in IP-CE (− 5.6%; CPP-IE: -9.6%; CPC-IE: -17.6%), but not in IP-IE	All *p* < 0.05
								↑* TTF in all groups	All *p* < 0.05
								*Anaerobic*	
								↑*PPO (IP-CE: 21.3% CPP-IE: 17.5%; CPC-IE: 28.1%; IP-IE: 13.5%) and MPO in all groups	All *p* < 0.05
								*Muscular* ↑* Muscle strength (IP-CE: 18.8%; CPP-IE: 20%; CPC-IE: 31.0%; IP-IE: 20.5%)	All *p* < 0.05
Amani-Shalamzari et al. [19]	N = 12 male futsal players, > 5 years Iran National league 2^nd^ Division	(1) BFR (2) CON	Proximal portion of thighs	(1) BFR 110% leg’s SBP. Increased 10% after every 2 sessions (2) No BFR Exact pressure values not specified	Pneumatic cuffs, inflated during exercise, deflated during rest periods	10 sessions across 3 weeks	3-a-side futsal game, 3-min activity, 2-min rest Sessions 1–3: 4 sets Sessions 4–7: 6 sets Sessions 8–9: 8 sets Session 10: 4 sets	*Muscular*	
								↑* Peak torque for knee extension and flexion, more in BFR (30.9% and 23.8%) than in CON (14.9% and 8.1%)	*p* = 0.01
								↑* iEMG of m.vastus lateralis, m. vastus medialis in both groups	*p* = 0.01
								↑* iEMG m.rectus femoris more in BFR than CON	*p* = 0.02
Amani-Shalamzari,et al. [20]								*Aerobic*	
								↑* $\dot{\mathrm{V}}{\mathrm{O}}_{2\mathrm{max}}$ and v $\dot{\mathrm{V}}{\mathrm{O}}_{2\mathrm{max}}$ in both groups (BFR:11.1% and 4.2%, CON: 6.8% and 2.2%)	Both* p* < 0.05
								↑*TTF and RE only in BFR (BFR:10.3% and -22.7% vs CON: 3.9% and − 4.2%)	Both *p* < 0.05
								*Anaerobic*	
								↑*PPO in both groups (BFR:12.7%, CON: 4.8%)	*p* < 0.05
								↑*MPO only in BFR group (BFR:12.2% vs CON: 1.7%)	*p* < 0.05
Christiansen, et al. [13]	n = 10 healthy, recreationally active men	(1) BFR leg (2) CON leg	Proximal portion of each thigh	(1) ~ 180 mmHg (2) No BFR	Pneumatic cuffs, inflated ~ 10-s prior to and deflated after each exercise bout	18 sessions, 3 times a week for 6 weeks	3 × 2-min cycling bouts, with 1-min rest. Total 3 sets, 2-min active recovery between sets 60%, 70%, 80% W_max_ in each set	*Muscular*	
								↑*iPPO in knee-extensor performance in BFR (23%) more than CON leg (12%)	*p* < 0.05
Christiansen, et al. [14]								↑*TTE in BFR (21%) more than CON leg (11%)	*p* = 0.001
Christiansen et al. [11]								↓*Relative intensity at 90% pre-training iPPO in BFR (18%) more than in CON(9%) leg	*p* = 0.002
Christiansen et al. [12]								↑*Power output at 25%iPPO in BFR(20%) more than CON(9%) leg	*p* = 0.017
Mitchell et al. [16]	n = 21 healthy males, competitive cyclists or triathletes	(1) BFR (2) CON	Proximal portion of each thigh	(1) ~ 120 mmHg (2) No BFR	Pneumatic cuffs, inflated within 25-s after each sprint, 2-min into rest	8 sessions, 2 sessions per week for 4 weeks	SIT: 30-s maximal effort sprint cycling, with 4.5 min recovery 4, 5, 6, 7 sets	*Aerobic* ↑* $\dot{\mathrm{V}}{\mathrm{O}}_{2\mathrm{max}}$ (5.9%) only in BFR	*p* = 0.04
								↑* Relative MAP (CON:1.5% and BFR: 3.5%), CP (CON:3.6% and BFR: 3.3%)	All *p* < *0.05*
								*Anaerobic*	
								↑*PPO (CON:5.2% and BFR: 7.2%) but no difference between them	Both *p* < 0.05
Elgammal et al. [17]	n = 24 highly trained university basketball players	(1) BFR (2) CON	Proximal region of thighs	(1) 100 mmHg, increased by 10 mmHg every session till 160 mmHg (2) No BFR	Pneumatic cuffs, inflated right before RS exercise	12 sessions, 3 sessions per week for 4 weeks	RST: 8 × maximal effort 15 m by 15 m sprints, with 20-s rest between reps 3 sets, with 4 min rest between sets	*Aerobic* ↑* $\dot{\mathrm{V}}{\mathrm{O}}_{2\mathrm{max}}$ in BFR (20.6%) more than CON (15%)	*p* = 0.04
								*Anaerobic*	
								No differences in changes in suicide run tests in both groups	*p* = 0.25
								*Muscular*	
								↑* 1RM Half-squat in BFR (17.8%) more than in CON (11.4%)	*p* = 0.02
								↑* 1RM Bench press in both groups (BFR: 14.1%, CON: 9.8%)	*p* < 0.05

↑*—significant increase, ↓*—significant decrease, Δ—changes, 1RM—1 repetition-maximum, AOP—arterial occlusion pressure, BFR—blood flow restriction, CON—control, CP—critical power, CPP-IE—constant partial occlusion pressure, increasing exercise intensity, CPC-IE—constant complete occlusion pressure, increasing exercise intensity, ${\mathrm{HR}}_{\mathrm{max}}$– maximal heart rate, iEMG—integrated myography, IP-CE—increasing occlusion pressure, constant exercise intensity, IP-IE—increasing occlusion pressure, increasing exercise intensity, MPO—mean power output p-BFR, practical blood flow restriction, PRV—peak running velocity, iPPO—incremental peak power output, PPO—peak power output, RFD—rate of force development, RE—running economy, RPE—rating of perceived exertion, SIT—sprint interval training, TT—time trial, TTE—time to exhaustion, TTF—time to fatigue, $\dot{\mathrm{V}}\mathrm{CO}_2$—carbon dioxide output, $\mathrm{VE}$—minute ventilation, ${\mathrm{VE}}_{\mathrm{max}}$– maximal exercise ventilation, $\dot{\mathrm{V}}{\mathrm{O}}_{2\mathrm{max}}$—maximal oxygen uptake, $\dot{\mathrm{V}}{\mathrm{O}}_{2\mathrm{max}}$ PRESS—maximal oxygen uptake from graded test with BFR, v $\dot{\mathrm{V}}{\mathrm{O}}_{2\mathrm{max}}$—running velocity at $\dot{\mathrm{V}}{\mathrm{O}}_{2\mathrm{max}}$,—${\mathrm{W}}_{\mathrm{max}}$maximal aerobic power

ª Research only included BFR groups, for exact BFR protocols please refer to the article

##### Participants

The total number of participants was 180, 128 (71.1%) were male, 52 (28.9%) were female. The number of participants per study ranged from 10 [11–14] to 32 [15]. Participants’ age range was between 18 and 39 years. Participants included in the studies were categorised based on a participant classification framework developed by McKay et al. [50]–which sorts participants into various tiers based on factors including sporting performance, training exposure, biometric attributes, and general fitness level. The types of participants ranged from untrained [18] to recreationally active [10–15, 32] and highly trained subjects [16, 17, 19, 20, 27]. All studies compared a BFR and CON (no BFR application) group except for the study of Amani-Shalamzari, et al. [15] which did not have a CON group but compared four training protocols of different exercise intensities and BFR occlusion pressures.

##### BFR Application

The site of BFR application was similar across all studies—at the proximal portion of each thigh. Methods of BFR application involved the use of pneumatic cuffs either through fixed occlusion pressures [11–18, 27] or percentage of systolic blood pressure (SBP) [19, 20] and use of elastic wraps through the practical BFR (p-BFR) method [10, 32]. Material of cuffs was either stated as nylon [11–14, 16, 27] or not mentioned [10, 15, 17–20, 32, 51]. Width of cuffs ranged from 5 to 13 cm [10–16, 19, 20, 27, 32] and diameter of cuffs ranged from 120 to 200 cm [15, 16, 19, 20, 27, 32]. Due to the differences in exercise protocols, the procedures in inflation/deflation of pneumatic cuffs and application of the elastic wraps (p-BFR) were varied. Most of the studies adopted an intermittent BFR application protocol whereby pressure was applied only during sets of work intervals and removed during rest/recovery periods [10–15, 18–20]. In SIT protocols, BFR was applied using pneumatic cuffs up to a pressure of ~ 130 mmHg, inflated within 15-s [27] and 25-s [16] after each sprint, and 2 min into recovery time before deflation. It was determined during pilot work that participants were not able to tolerate BFR throughout the entire 30-s all-out interval, even at moderate cuff pressures of 100 mmHg [27]. In the other two studies which also adopted sprint-based type exercise, continuous BFR application was used with pressure applied throughout the entire exercise duration [17, 32].

#### Aerobic Performance

Out of seven studies which investigated changes in aerobic performance, five studies reported positive outcomes [10, 15–17, 20, 27] and one study reported no additive outcomes [18] of including BFR into HIIT exercise protocols. The seventh study by Amani-Shalamzari et al. [15] did not have a CON (non-BFR) group, but they likewise observed positive outcomes in aerobic performance across all BFR groups and provided evidence on how differing progressions of occlusion pressures and exercise intensities during a BFR + HIIT exercise intervention may affect chronic performance adaptations.

##### Maximal Aerobic Capacity

Two studies [16, 27] observed a ~ 4.5% and ~ 5.9% increase in maximal oxygen uptake ($\dot{\mathrm{V}}{\mathrm{O}}_{2\mathrm{max}}$) of trained male cyclists only in the BFR group—this was after 8 sessions (2 sessions per week across 4 weeks) of SIT (4 to 7 sets of 30-s maximal sprint cycling and 4.5-min recovery). Earlier work attributed performance improvements after SIT to peripheral adaptations—i.e. an increase in arterial-venous oxygen difference, rather than an increase in cardiac output [52, 53]. However, Mitchell et al. [16] conducted muscle biopsies on the trained cyclists and noticed that peripheral qualities like skeletal muscle capillary density or mitochondrial protein content were unchanged, and hence concluded that the increase in $\dot{\mathrm{V}}{\mathrm{O}}_{2\mathrm{max}}$ in the BFR group was instead attributable to central adaptations (e.g. cardiac output). The differing conclusions among studies may be attributed to the methods of investigation (acetylene non re-breathing techniques to measure cardiac output versus muscle biopsy) and future studies should clarify the impact of HIIT on central versus peripheral adaptations [16, 53], while using standardised methods for clearer comparisons.

The combination of BFR + RST also seemed to be significantly more effective than RST alone in increasing $\dot{\mathrm{V}}{\mathrm{O}}_{2\mathrm{max}}$ (20-m shuttle run test) in highly trained university basketball players (BFR: +  ~ 20.6% vs CON: +  ~ 15%) [17]. This was achieved after 12 sessions (3 sessions per week for 4 weeks) of RST (3 sets of 8 repetitions of maximal effort 15 m by 15 m sprints with 20-s recovery).

In the study of Paton et al. [10], investigators observed significant improvements in $\dot{\mathrm{V}}{\mathrm{O}}_{2\mathrm{max}}$ in both groups after 8 sessions (2 sessions per week for 4 weeks) of a running intervention (2–3 sets of 5–8 repetitions of 30-s running at 80% peak running velocity (PRV), 30-s rest). Despite a greater percentage increase in $\dot{\mathrm{V}}{\mathrm{O}}_{2\mathrm{max}}$ in the BFR (6.4%) than CON (4.0%) group, the difference between the groups was not significant (*p*-value = 0.33). Likewise, Amani-Shalamzari et al. [20] found that inclusion of BFR in SSG training of male futsal players (10 sessions across 3 weeks of 3-a-side high-intensity futsal game, 4–8 sets of 3-min activity, 2-min rest) resulted in a significant increase in $\dot{\mathrm{V}}{\mathrm{O}}_{2\mathrm{max}}$ (treadmill test), with a non-significant trend toward an increase over the CON group (BFR: ~ 11.1%; CON: ~ 6.8%, *p*-value between groups: 0.11).

One study [18] reported no differences in the $\dot{\mathrm{V}}{\mathrm{O}}_{2\mathrm{max}}$ of untrained subjects after 18 sessions (3 sessions per week for 6 weeks) of training. This could be due to differences in intensities of the 2-min intervals—intensity of 90%$\dot{\mathrm{V}}{\mathrm{O}}_{2\mathrm{max}}$ for CON group vs 90%$\dot{\mathrm{V}}{\mathrm{O}}_{2\mathrm{max}}$ PRESS (intensity derived after a similar graded exercise test with BFR cuffs) for BFR group.

In the study of Amani-Shalamzari et al. [15], 32 healthy active collegiate females were randomly allocated into four BFR groups—increasing BFR pressure with constant exercise intensity (IP-CE), constant partial BFR pressure with increasing exercise intensity (CPp-IE), constant complete BFR pressure with increasing exercise intensity (CPc-IE) and increasing BFR pressure with increasing exercise intensity (IP-IE). Exercise intervention lasted for 4 weeks (total of 12 sessions, 3 sessions per week for 4 weeks) and consisted of 10 sets of 2-min running with 1-min recovery except for IP-IE group (10, 8, 6 and 5 sets in each week). All groups observed significant increases in $\dot{\mathrm{V}}{\mathrm{O}}_{2\mathrm{max}}$ after the training intervention.

As observed, significantly positive improvements in $\dot{\mathrm{V}}{\mathrm{O}}_{2\mathrm{max}}$ were evident with the addition of BFR into sprint-based protocols of SIT [16, 27] and RST [17]. However, these differences in improvements were not reflected with the inclusion of BFR into submaximal aerobic [10] and SSG [20] training protocols although non-significant trends of greater improvements in BFR were observed.

##### Maximal Aerobic Power, Critical Power, Velocity at $\dot{\mathrm{V}}{\mathrm{O}}_{2\mathrm{max}}$, and Peak Running Velocity

Six out of seven studies assessed and compared changes in measurements of maximal aerobic function after BFR + HIIT interventions. In Taylor et al. [27], there were no significant improvements of MAP in trained cyclists in BFR (2.9–4.4%) or CON (0.2–0.3%) groups.

Four studies observed positive improvements in BFR and CON groups, but with no differences between them [10, 16, 18, 20]. The study by Mitchell et al. [16] which adopted a similar BFR + SIT protocol as Taylor et al. [27], saw improvements in CP and relative MAP with training, but without any differences between CON and BFR groups. Amani-Shalamzari et al. [20] also observed improvements in velocity at $\dot{\mathrm{V}}{\mathrm{O}}_{2\mathrm{max}}$ (v $\dot{\mathrm{V}}{\mathrm{O}}_{2\mathrm{max}}$) in both BFR (~ 4.2%) and CON (~ 2.2%) groups after SSG training. In untrained subjects, although there were no observed changes in $\dot{\mathrm{V}}{\mathrm{O}}_{2\mathrm{max}}$, MAP was increased in both the BFR (~ 25%) and CON (~ 15%) training interventions [18]. Likewise, the study by Paton et al. [10] demonstrated that PRV and incremental run time improved in both BFR (~ 3.6% and ~ 6.1%, respectively) and CON group (~ 1.5% and ~ 2%, respectively). In Amani-Shalamzari et al. [15]’s research, they observed that v $\dot{\mathrm{V}}{\mathrm{O}}_{2\mathrm{max}}$ also increased significantly in all BFR groups (no CON group) despite different BFR + LT protocols.

Although the inclusion of BFR into HIIT was observed to promote greater adaptations in maximal aerobic function [10, 16, 18, 20], more evidence and clarification is needed. It is important to standardise the study designs—type of HIIT protocol, total duration of intervention, exercise mode, type of participants, etc.—to better understand how the interaction of BFR and HIIT can affect chronic adaptations in maximal aerobic function.

##### Time Trial Performance, Submaximal Tests, Time to Fatigue Tests, and Running/Cycling Economy

Five studies investigated aerobic performance outcomes beyond $\dot{\mathrm{V}}{\mathrm{O}}_{2\mathrm{max}}$ and maximal aerobic function [10, 15, 18, 20, 27]. In trained male cyclists undergoing eight sessions of SIT, although $\dot{\mathrm{V}}{\mathrm{O}}_{2\mathrm{max}}$ improved in the BFR + SIT group but not in the CON group, neither group achieved a faster time in a 15 km cycling time trial (TT) [27]. It was suggested that a 15 km TT (~ 20–25 min) may have lacked sensitivity to reflect the improvement associated with an increase in $\dot{\mathrm{V}}{\mathrm{O}}_{2\mathrm{max}}$—TT distance must be considered during evaluation of self-paced performance, i.e. exercise would predominantly be constrained by peripheral fatigue during short, high-intensity (~ 6 min) TTs or central fatigue during long, lower-intensity (> 30 min) TTs [27, 54].

In the study of Keramidas et al. [18], cycling $\dot{\mathrm{V}}{\mathrm{O}}_{2}$ was significantly reduced (~ 78% to ~ 72%) during a 6-min submaximal aerobic test at a fixed workload (80%$\dot{\mathrm{V}}{\mathrm{O}}_{2\mathrm{max}}$ of pre-test) in both groups. Moreover, time to fatigue (TTF) at 150% MAP was significantly improved without any differences between groups. The inclusion of BFR into SSG was observed to significantly improve both TTF at 100%v $\dot{\mathrm{V}}{\mathrm{O}}_{2\mathrm{max}}$ and running economy (RE) as compared to SSG alone (BFR: ~ 10.3% and − ~ 22.7% vs CON: ~ 3.9% and − ~ 4.2%) [20]. Although there were no TTF measurements, Paton et al. [10] also observed that RE was only improved in the BFR + ST group (− ~ 6.7%) but not the CON (+ ~ 2.1%) group. The small additional enhancements between BFR and CON groups in various aerobic tests (e.g. $\dot{\mathrm{V}}{\mathrm{O}}_{2\mathrm{max}}$, TTF, RE, PRV or v $\dot{\mathrm{V}}{\mathrm{O}}_{2\mathrm{max}}$) in these two studies [10, 20] were interpreted to be due to an increase in internal training load (i.e. exercise HR, increase in bLa, etc.) during training sessions. It was suggested that the increased training load led to adaptations in the anaerobic and muscular systems—increase in muscle cross-sectional area, activation and strength, delay in recruitment of type II muscle fibres (thus delay in rise of bLa levels), increases in muscle buffering capacity and higher lactate tolerance [8, 55]. In Amani-Shalamzari et al. [15], all BFR groups experienced significant improvements in TTF at 100%v $\dot{\mathrm{V}}{\mathrm{O}}_{2\mathrm{max}}$, but RE was only significantly improved in the IP-CE (− ~ 5.6%), CPp-IE (− ~ 9.6%) and CPc-IE (− ~ 17.6%) groups and not in the IP-IE group (− ~ 6.3%).

The current pool of evidence indicates that while maximal effort BFR + SIT may not improve cycling TT performance, the implementation of BFR into submaximal effort and mixed intensities HIIT, e.g. ST, LT and SSG, may likely enhance improvements in exercise economy and TTF during both submaximal and supramaximal aerobic exercise due to an increased internal load during training sessions [10, 15, 18, 20].

#### Anaerobic Performance

Out of six studies which measured anaerobic performance, five reported positive outcomes from pre- to post-tests for anaerobic performance in either both CON and BFR groups [16, 27, 32] or in all BFR groups [15], and one study reported no differences after the exercise intervention [17]. Differences in improvements between CON and BFR groups were observed in two studies [20, 32], while differences in improvements between BFR groups were observed in one study [15].

##### Peak Power Output, Mean Power Output, and Sprint Speed

Elgammal et al. [17] reported that both BFR and CON groups did not improve their anaerobic capabilities, measured as performance on a basketball-specific suicide test, after 12 sessions of RST. In the studies of Taylor et al. [27] and Mitchell et al. [16], after eight sessions of SIT, both BFR and CON groups improved in the cycling sprint PPO, but without any between-group differences (BFR: ~ 6.8% and ~ 7.2% vs. CON: ~ 6.8% and ~ 5.2%, respectively). However, the use of BFR in submaximal effort SIT elicited significantly greater improvements in maximal sprint speed than CON—mean 100 m sprint times were reduced by 0.38-s (~ 3%) in BFR vs 0.16-s (~ 1.3%) in CON group [32].

In Amani-Shalamzari et al. [20], after 10 sessions of SSG, futsal players in the BFR group exhibited significantly greater improvements in mean power output (MPO) on a Wingate test as compared with the CON group (BFR: ~ 12.2% vs CON: ~ 1.7%). There was also a trend toward greater improvement in PPO in the BFR group (BFR: ~ 12.7% vs CON: ~ 4.8%). The study of Amani-Shalamzari et al. [15] showed that all four BFR + LT protocols were effective in improving the PPO and MPO of female students in a Wingate test, but CPc-IE was significantly superior in promoting anaerobic adaptations in PPO (as compared to IP-IE group) and MPO (as compared to IP-IE and CPp-IE groups).

Sprint-based protocols which require all-out maximal efforts do not appear to induce additional benefits when BFR was administered [16, 27]. This contrasts with the application of BFR + submaximal sprint efforts (60–70% of maximal sprint speed), which improve maximal sprint speed more than that of CON [32]. The addition of BFR to HIIT protocols of SSG and LT was beneficial in promoting greater anaerobic adaptations of MPO and PPO as compared to HIIT alone.

#### Muscular Performance

Of eight studies which investigated muscular performance, all reported positive outcomes from pre- to post-tests in muscular performance in both BFR and CON groups [11–14, 17, 19, 32], and with multiple BFR groups [15]. The seven studies with both BFR and CON groups also reported significant benefits in certain muscular performance parameters after BFR + HIIT exercise intervention as compared to just HIIT exercise itself. Note that in the studies by Christiansen and colleagues [11–14], participants underwent training with BFR on one leg and no BFR on the other (CON). Although there was no CON group in the study by Amani-Shalamzari et al. [15], investigators found that the type of BFR + HIIT protocol prescribed had a huge impact on the extent of muscular performance gains.

##### Muscular Strength and Power

After 12 sessions of submaximal effort sprint training, it was observed that rate of force development (RFD) during a leg press test was significantly improved with BFR (25%) compared with a CON group (1.7%) [32]. Behringer and colleagues [32] mentioned that implementing BFR with submaximal effort sprint training could recruit more type II muscle fibres, and a higher metabolite accumulation in blood flow restricted muscles would cause a reflex inhibition of alpha motoneurons via group III and group IV afferents, resulting in increased type II motor unit activation to maintain force output [56]. The knee extension and flexion test on the isokinetic dynamometer conducted by Amani-Shalamzari et al. [19] also showed greater improvements in peak torque in BFR (~ 30.9% and ~ 23.8%) than in CON (~ 14.9% and ~ 8.1%, respectively) after 10 sessions of SSG training. Furthermore, higher internal training load (HR and bLa) was observed with the addition of BFR into SSG during the first training session—this led to significantly higher levels of testosterone, growth hormone (GH) and testosterone to cortisol ratio in BFR than CON (~ 54.2%, ~ 28.8% and ~ 30.4%, respectively). With greater physiological stress, elevated testosterone and GH levels, it was likely that participants in the BFR group experienced greater training adaptations as they recovered from each SSG session, leading to greater improvements in muscular, aerobic, and anaerobic performances compared to CON [19, 20].

In an incremental knee extensor performance test (iPPO), it was observed that the power output of the BFR-leg (23 ± 9%) improved significantly more than the CON-leg (12 ± 6%) within subjects after 18 sessions of training [11–14]. It was also reported that the relative intensity at 90% pre-training iPPO was reduced to a significantly greater extent in the BFR-leg (~ 18%) than in the CON-leg (~ 9%), and power output at 25% iPPO was ~ 11% greater in the BFR-leg than in the CON-leg after training. The authors suggested that the improved muscular performance in the BFR-leg stemmed from several chronic physiological adaptations: (1) enhanced potassium ion regulation [14], (2) higher thigh net glucose uptake [13], (3) increases in thigh oxygen (O_2_) delivery, uptake and femoral artery diameter [11] and (4) increased capacity for hydrogen ion exchange via lactate dependent hydrogen ion transport and blood hydrogen ion buffering capacity [12].

Elgammal et al. [17] reported that BFR + basketball-specific RST aided in the improvements of 1-repetition-max half-squat over just RST itself (BFR: ~ 17.8% vs CON: ~ 11.4%). Of four different BFR-LI protocols in Amani-Shalamzari et al. [15], constant complete occlusion with increasing exercise intensity (CPc-IE) was the most potent for improving muscular strength in the legs (CPc-IE: ~ 31.0% vs IP-CE: ~ 18.8% vs CPp-IE: ~ 20.0% vs IP-IE: ~ 20.5%).

Notably, the application of BFR into HIIT interventions amplified the improvements made in muscular strength and power assessed via various muscular strength tests [11–14, 17, 19, 20, 32]—these results were consistent throughout most types of HIIT protocols, i.e. LT, SSG, submaximal effort SIT and RST.

##### Muscular Hypertrophy, Endurance, and Activation

Behringer et al. [32] also reported that the muscle thickness of the rectus femoris with BFR (~ 5.7%) training increased significantly compared with CON (~ 0.4%), and there was also a non-significant trend towards a difference in biceps femoris muscle thickness (BFR: ~ 5.7% vs CON: ~ 2.7%). This aligns with previous literature which indicated an increase of 4–7% volume of thigh muscles after 3 weeks of BFR low-intensity walk training [57].

In terms of muscular endurance, it was also observed that BFR + LT training substantially increased time to exhaustion during exhaustive exercise (BFR-leg lasted 11 ± 5% longer than CON-leg) [14].

The improvements in knee flexion and extension tests in Amani-Shalamzari et al. [19] were accompanied by an increase in integrated electromyography (iEMG) signals in the vastus lateralis, vastus medialis and rectus femoris of both groups, but with the improvements of iEMG rectus femoris being significant more in the BFR than in the CON group (~ 60.5% vs ~ 19.0%, respectively). This was deemed as a result of increased metabolite accumulation during BFR training, which led to greater recruitment of type II fibres through an increase in motor units engaged [58–60].

Present evidence affirms that BFR elicits greater improvements in interrelated muscular parameters, i.e. strength, power, endurance, hypertrophy, and muscle activation.

## Discussion

The aim of this systematic review was to evaluate the available scientific literature regarding the acute performance, metabolic (vascular, oxygenation, biochemical and molecular), neuromuscular and perceptual responses, as well as chronic performance (aerobic, anaerobic and muscular) adaptations of the various BFR + HIIT protocols. This section presents and discusses the consensus summary derived from the results.

### Acute Responses of BFR + HIIT

**Fig. 2:**
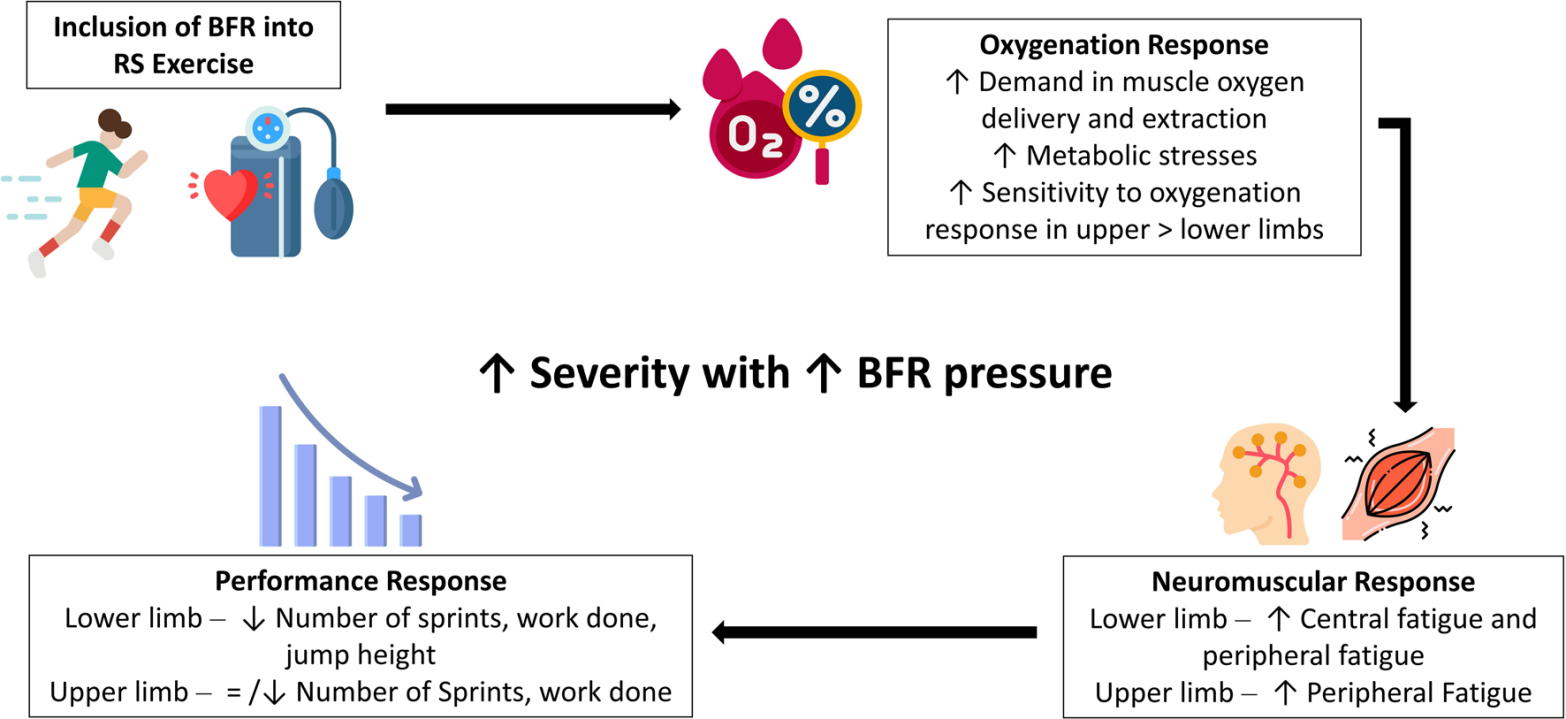
Flowchart of main acute responses when including blood flow restriction (BFR) into repeated sprint (RS) exercise. The implementation of BFR into RS exercise amplifies oxygenation responses—there is higher demand for muscle oxygen delivery and extraction which causes an increase in metabolic stresses. This leads to comparatively quicker onset of neuromuscular fatigue and thus decrease in RS performance (i.e. number of sprints, work done, and jump height). The decrease in RS performance is more evident in BFR + lower-limb than BFR + upper-limb-based RS exercise. This is due to the higher sensitivity to oxygenation and greater hyperaemic effect in upper limbs as compared to lower limbs, which allow upper limbs to react better to the increased oxygen demand caused by BFR. As such, any decrease in upper-limb RS performance is likely to be caused by increase in peripheral fatigue induced by BFR. On the other hand, the decrease in lower-limb RS performance is possibly induced by increase in central and peripheral fatigue brought about by BFR. Lastly, the increase in BFR pressure will lead to an increased severity of oxygenation responses, higher neuromuscular fatigue and thus greater decrease in performance response

The six studies that investigated acute responses of BFR + HIIT mainly adopted the use of BFR with maximal effort sprint-based exercise either in the form of RST [26, 28–31] or SIT [27]. Evidence from the current literature suggests that there are indeed differences when BFR was included into sprint-based exercise: (1) including BFR into sprint-based protocols may accelerate fatigue mechanisms associated with RS exercise protocols impairing performance, (2) there are differences in upper-limb and lower-limb (arm vs leg cycling) BFR + RS responses, and (3) high occlusion pressures may not be suitable for sprint-based training method. Firstly, for lower-limb RST, it was evident that performance measures of total number of sprints and work done were negatively affected when BFR was applied during exercise [28–30]. This was different from those of upper-limb RST where there were mixed results—two studies observed no significant performance impairments [30, 31], and one showed significant performance impairment in number of sprints performed [26]. BFR may have accelerated neuromuscular fatigue during RST through both peripheral and central mechanisms—due to the accentuation of vascular regulation of blood flow and thus greater changes in oxygenation responses (particularly at the muscular level, refer to Fig. 2), as well as a possible increase in the severity of metabolic processes (intracellular accumulation of hydrogen ions, reduction in bLa removal, and phosphocreatine breakdown at the muscular level) (refer to the Neuromuscular Responses section).

Secondly, the disparity in performance responses observed between upper and lower-limb BFR + RS cycling could likely be the consequence of varying metabolic responses that occurred within the arm and leg musculature [30]. As mentioned in  the Metabolic Responses section, the smaller musculature of the arms may be more sensitive to oxygenation and have a greater hyperemic effect than the larger leg musculature. This would have affected the overall oxygen delivery and uptake of the working muscles and hence performance outcomes (refer to Fig. 2).

Finally, high occlusion pressures were observed to limit performance and may not be suitable for sprint-based exercise. For leg-cycling BFR + RST exercise, a higher occlusion pressure of 60%PEP vs 45%PEP was observed to cause more severe oxygenation responses, impairing participants’ ability to fully exhaust the cardiovascular and respiratory system [29]. Similarly, during cycling BFR + SIT which included maximal sprints of 30 s, investigators observed that in the pilot tests conducted, participants were unable to sprint maximally even with a reduced occlusion pressure of ~ 100 mmHg applied at the start of exercise, and thus had to alter their protocol accordingly [27].

### Chronic Effects of BFR + HIIT

**Fig. 3:**
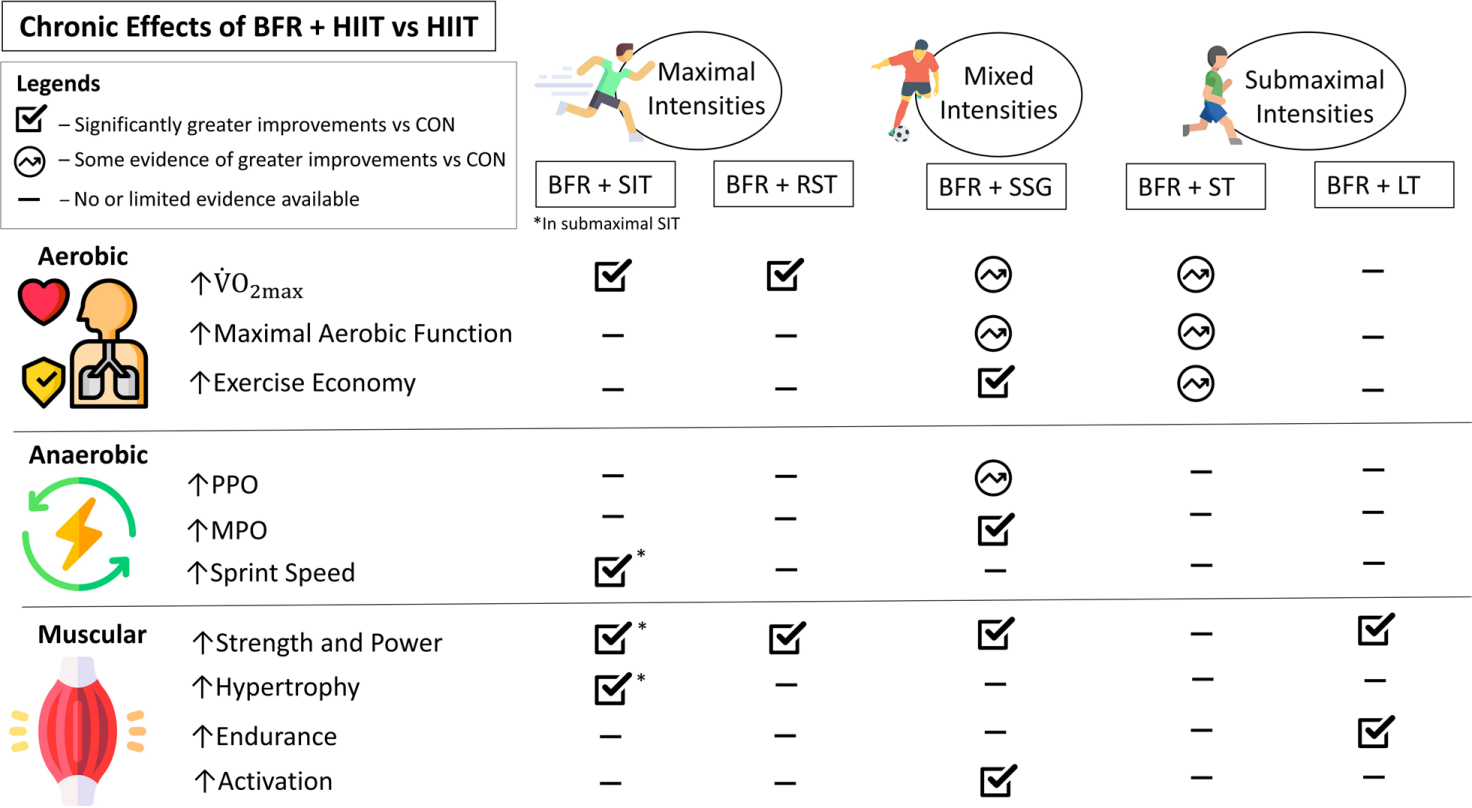
Overview of the chronic effects (aerobic, anaerobic, and muscular adaptations) of implementing blood flow restriction into high-intensity interval training (BFR + HIIT) vs HIIT (CON) based on present evidence. The terms ‘maximal’ and ‘submaximal’ relate to exercise intensities based on the level of exertion/effort. The symbol ‘  ’ signifies significantly greater improvements vs CON, ‘  ’ signifies some evidence of greater improvements vs CON, while ‘—' signifies no/insufficient evidence to provide a conclusion. BFR + sprint protocols, including sprint interval training (SIT) and repeated sprint training (RST) improve maximal aerobic capacity ($\dot{\mathrm{V}}{\mathrm{O}}_{2\mathrm{max}}$). BFR + submaximal effort SIT improves running sprint speed, muscular strength, power, and hypertrophy. There are greater improvements in strength and power after BFR + RST. For BFR + small-sided games (SSG), there are greater improvements in exercise economy, mean power output (MPO), muscular strength, power, and activation, as well as some evidence of greater improvements in $\dot{\mathrm{V}}{\mathrm{O}}_{2\mathrm{max}}$, maximal aerobic function and peak power output (PPO). BFR can also be paired with submaximal effort HIIT methods like short intervals (ST) and long intervals (LT). For BFR + ST, there is some evidence of a greater improvement for $\dot{\mathrm{V}}{\mathrm{O}}_{2\mathrm{max}}$, maximal aerobic function and exercise economy. For BFR + LT, muscular parameters of strength, power and endurance are significantly improved

The weight of evidence suggests that implementing BFR into HIIT can enhance chronic performance adaptations in aerobic and muscular parameters, whereas improvements in anaerobic components may only be limited to the inclusion of BFR in submaximal effort exercise interventions. Figure 3 presents a summary of the chronic effects of BFR + HIIT vs solely HIIT.

From the perspective of aerobic adaptations (refer to the Aerobic Performance section), addition of BFR into sprint-based (SIT or RST) protocols elicited significantly greater improvements in $\dot{\mathrm{V}}{\mathrm{O}}_{2\mathrm{max}}$ as compared to CON groups [16, 17, 27]. Given that past research on sprint-based protocols demonstrated that SIT and RST interventions promote improvements in $\dot{\mathrm{V}}{\mathrm{O}}_{2\mathrm{max}}$ via peripheral adaptations like mitochondrial biogenesis and angiogenesis [52, 61], it was unexpected that Mitchell and colleagues [16] did not observe any changes in capillary density or any significant changes at the muscular level in both SIT groups. They thus postulated that central adaptations (i.e. increased cardiac output) might have contributed to the increased $\dot{\mathrm{V}}{\mathrm{O}}_{2\mathrm{max}}$ only in the BFR + SIT group. However, participants in both SIT studies [16, 27] were well-trained cyclists, and thus a similar intervention protocol may elicit different adaptations in less trained individuals.

Inclusion of BFR into mixed intensities (SSG), or submaximal aerobic (ST) protocols resulted in greater improvement in $\dot{\mathrm{V}}{\mathrm{O}}_{2\mathrm{max}}$ compared with CON but these differences were non-significant. Moreover, trends in greater improvements were also observed in other indicators of aerobic performance including RE, PRV and TTF tests [10, 20]. The authors proposed that these changes were due to increases in internal training load (i.e. HR and bLa) imposed by BFR during HIIT, which caused higher metabolic stresses and possibly promoted peripheral adaptations to a greater extent than CON in terms of muscle buffering capacity, lactate tolerance and muscular strength. The only study which did not exhibit additive aerobic adaptations of BFR + HIIT was that of Keramidas et al. [18]. As mentioned in the Maximal Aerobic Capacity section, in this study, the absolute training intensities for both BFR + HIIT and CON group were different—prescription of exercise intensity for BFR group was based upon a graded exercise test with BFR, rather than a normal graded exercise test in the case of the CON group—thus comparisons may not be valid.

Regarding chronic anaerobic adaptations (refer to  the Anaerobic Performance section), BFR did not seem to elicit any additive effects in basketball-specific suicide test [17] or cycling sprint PPO [16, 27] as compared to CON, when maximal sprint-based protocols like RST or SIT were adopted. However, BFR was effective in elevating 100 m sprint speed [32] and MPO during Wingate tests [20] after training interventions which include submaximal (70% of fastest sprint speed) SIT or SSG (mixed intensities), respectively. The anaerobic improvements made after implementing BFR into submaximal/mixed intensity protocols may be a consequence of increased neural involvement and muscle activation—as BFR was shown to increase iEMG during lower intensity exercise [59, 60]. This is in contrast to all-out or maximal exercise, where BFR seems to impair, rather than increase muscle activation levels as depicted in the acute BFR + RST studies [26, 29].

An undisputed, distinctive advantage of implementing BFR into HIIT exercise is the enhanced muscular adaptations that are achieved after a block of training intervention. All seven BFR + HIIT studies which measured muscular performances revealed significant improvements with BFR compared to CON group (refer to the Muscular Performance section) in one or more areas of muscular strength, power, activation, hypertrophy, and endurance. These studies encompassed BFR + HIIT interventions of RST [17], submaximal effort SIT [32], submaximal aerobic intervals [11–15] and SSG [19]. The mechanisms responsible for these improvements in muscular performances with BFR are suggested to include: (1) increased metabolic stresses and thus internal load of exercise leading to higher type II motor unit activation, elevated growth hormone concentration, potassium ion regulation, thigh net glucose uptake and muscle buffering capacity [12–14, 19, 32], and (2) limiting O_2_ delivery to working muscles which leads to increased vascular regulation and improved oxygen uptake [11].

### Standardisation of BFR + HIIT Intervention Methods

In studies included in this review, a plethora of BFR methodologies were adopted—from the various types of BFR application, i.e. SBP, PEP, AOP, fixed occlusion pressure or p-BFR, to the approaches in application of pressure, i.e. continuous (applied throughout exercise duration) or intermittent (applied during exercise and removed during recovery). As the different types of HIIT protocols (e.g. SIT, RST, LT, ST or SSG) also consist of a wide spectrum of intensity profiles, sport scientists may have to alter and modify BFR methodologies for each mode of training. For example, Taylor et al. [27] found that even at an occlusion pressure of 100 mmHg, cyclists could not tolerate a 30 s maximal effort sprint and thus adjusted to applying BFR pressure (130 mmHg) within 15-s after each 30 s maximal sprint for a period of 2 min into a 4.5 min recovery period. However, the limitation was that the percentage of blood flow restriction (at 130 mmHg) of each participant was not quantified [27]. Considering individual variations in maximal limb occlusion pressure, fixed occlusion pressure, SBP or p-BFR methods may pose safety risks, as a predetermined occlusion pressure may result in varying degrees of BFR stimulus imposed to different participants. Therefore, to ensure a consistent and safe BFR stimulus while exercising, it is recommended that individualised limb occlusion pressure methods, i.e. AOP and PEP, through the use of pneumatic cuffs should be employed by sports practitioners [7]. The standardisation in the use of individualised BFR pressures would subsequently allow a basis of comparison between future investigations into BFR + HIIT—e.g. acute responses of intermittent vs. continuous BFR, differences in higher vs lower %AOP during exercise, or chronic effects of various BFR + HIIT training interventions, etc.

The extent of chronic performance adaptations can be significantly affected by the prescription of BFR methodology and HIIT exercise intensities. In the study of Amani-Shalamzari et al. [15], investigators demonstrated that magnitude of BFR pressure and exercise intensities did alter aerobic, anaerobic, and muscular gains derived by collegiate women after 12 sessions of BFR + LT—complete occlusion pressure and progression of exercise intensities throughout the weeks were shown to be the most effective. Moreover, pressure was only applied intermittently during exercise work bouts and removed during rest periods, which would possibly reduce swelling and perception of pain but still allow for a similar amount of muscle fatigue to occur [62, 63].

It is also critical to note that in order to elicit an increased training stimulus throughout the training sessions, there has to be a progression in BFR + HIIT sessions—either through increase in BFR stimulus (occlusion pressure), exercise intensity, or duration [10, 15–20, 27]. However, given the many training variables associated with BFR + HIIT, there is a need for future investigations to explore, recommend and provide standardised guidelines in BFR methodology. These should be targeted at recommending exercise intensity progressions for each type of HIIT protocol, mode of training and specific muscle groups (i.e. upper body vs lower body).

### Limitations of Research to Date

It is important to note that there are limitations within the research articles included in this systematic review (refer to the Results section). These were reflected in the quality assessment scores (PEDro scale) of the articles– 6 out of 18 studies scored 5 (‘Fair’), while 12 out of 18 studies scored 6 (‘Good’), out of a possible 10 points (Tables 1 and 2). Although all studies except for one [16] did provide evidence of random allocation of conditions or groups (criteria 2 of PEDro scale) either in the abstract or methodology sections, the randomisation process was not clearly described.

Moreover, it was clear that all the articles did not meet criteria 3 (‘concealed allocation’), 5 (‘blinding of subjects’), 6 (‘blinding of therapists’) and 7 (‘blinding of assessors’) —all four criteria may have threatened the internal validity of the findings. Future research should make every effort to meet criteria 3, which is possible to achieve, while criteria 5, 6 and 7 may be more challenging due to the application of BFR cuffs/wraps as the 'treatment' condition/group, which is difficult to conceal from subjects, therapists, and assessors. One way to achieve criteria 6 and 7 would be to have different individuals assessing the outcome of the intervention from those delivering it but this may be challenging for some research groups.

Another possible limitation of this research would be the inclusion of research articles with BFR + HIIT exercise intensities of > 60% $\dot{\mathrm{V}}{\mathrm{O}}_{2\mathrm{max}}$ [22, 23] and > 80%$H{\mathrm{R}}_{\mathrm{max}}$ [24]. Exercise intensities at the range of 60–80% $\dot{\mathrm{V}}{\mathrm{O}}_{2\mathrm{max}}$ in Christiansen et al. [11–14], 60–85%$\dot{\mathrm{V}}{\mathrm{O}}_{2\mathrm{max}}$ in Amani-Shalamzari et al. [15], and 80% $\dot{\mathrm{V}}{\mathrm{O}}_{2\mathrm{max}}$ in Paton et al. [10], could be viewed as more moderate than high intensity exercise. However, due to lack of a clearly agreed categorial definition of HIIT that is widely accepted, the authors based the inclusion criteria on current expert definitions [22–24] which tend to capture a broader range of BFR + HIIT studies. Although this provides the advantage of a full review of available studies, there is a potential of incorrectly categorising the intensity of exercise.

### Recommendations for Future Research

The investigations which reported findings on acute responses of BFR + HIIT were only limited to maximal sprint-based protocols (SIT and RST). As these sprint-based HIIT modalities are of an all-out, maximal intensity and highly anaerobic nature, the acute responses (metabolic, neuromuscular, perceptual, etc.) observed may be very different from that of submaximal exercise [63–66]. Hence, there is a need to compare acute responses of other BFR + HIIT protocols, e.g. submaximal exercise intervals at different ranges of intensities—which will provide valuable insight into the underlying mechanisms behind possible chronic physiological adaptations after a block of BFR + HIIT intervention.

In contrast, investigations on the chronic effects of BFR + HIIT solely explored lower-limb (cycling, running, repeated suicide sprints), but not upper-limb-based exercise modalities (e.g. arm cycling, rowing, etc.). As observed in Willis et al. [30], although similar BFR + RST cycling protocols were conducted on different muscle groups (arms vs legs) within the same participants, acute vascular and oxygenation responses significantly differed—this implies that the long-term adaptations induced by the same training method on different body parts may be vastly different. Moreover, differences in exercise modes and movements (e.g. cycling vs. running vs. rowing vs other sport-specific modalities) may affect acute responses during exercise due to variations in oxygen (${\mathrm{O}}_{2}$) uptake kinetics, peak ${\mathrm{O}}_{2}$ consumption, skeletal muscle ${\mathrm{O}}_{2}$ capacity and neuromuscular responses, thus leading to varying magnitudes of physiological adaptations [67]. Therefore, it is critical to examine both short and long-term responses of BFR + HIIT protocols specific to a type of exercise.

Although it is evident that including BFR into HIIT does promote positive chronic adaptations as compared with solely HIIT (Fig. 3), it is of necessity to conduct more research to investigate into the effects of different BFR + HIIT protocols on the three main parameters of physiological adaptations—aerobic, anaerobic, and muscular—to present new evidence or substantiate current findings.

An interesting aspect of this review was the examination of the application of lower-limb BFR into intermittent sport-specific methods like futsal SSG [19, 20], basketball and badminton RST [17, 28]. Based on these studies, BFR seemed to amplify participants’ internal training load and promoted positive training adaptations in both SSG [19, 20] and RST [17] compared to those exercising without BFR. More research investigations are to be conducted to confirm the effectiveness of occlusion combined with these exercise modalities. There are also a variety of factors to consider in the application of BFR into sport-specific HIIT modalities. These include: (1) which types of sport-specific HIIT methods will benefit from BFR, e.g. lower-limb BFR on change of direction movements, upper-limb BFR on ball throwing or racket swinging movements in both field and racket sports, (2) whether the attachment of occlusion cuffs on the upper or lower limbs may impede and alter biomechanical characteristics of sport skills, (3) how occlusion may affect perceptual response of discomfort and RPE during sport-specific HIIT, and (4) windows of opportunity to adopt BFR + HIIT methods in the periodisation of intermittent sport athletes.

## Conclusion

This review is the first to focus on the acute performance, metabolic (vascular, oxygenation biochemical and molecular), neuromuscular and perceptual responses, as well as chronic performance (predominantly aerobic, predominantly anaerobic and muscular) effects of BFR + HIIT exercise protocols. Studies investigating the acute responses of BFR + HIIT have primarily included BFR into maximal sprint-based protocols like RST and SIT. The localised hypoxia brought about by BFR challenges the metabolic processes (vascular adaptation and oxygenation responses) during high-intensity RS exercise, accelerating central and peripheral fatigue mechanisms, and leading to increased physiological stresses for the individual. The analysis of the literature exploring chronic effects of BFR + HIIT confirms that BFR does provide an additive physiological training effect to HIIT protocols, especially for aerobic and muscular parameters. Anaerobic components were only improved after implementing BFR in sub-maximal HIIT exercise interventions. Due to the large variability in permutations of BFR methodologies and HIIT exercise types, there is a necessity for future research to explore and recommend standardised BFR guidelines for each HIIT exercise type to ensure positive physiological outcomes for targeted populations.

## Data Availability

All data generated or analysed during this study are included in this published article [and its Additional files].

## Abbreviations

BFR: Blood flow restriction
HIIT: High-intensity interval training
LT: Long-interval training
ST: Short-interval training
SIT: Sprint interval training
RST: Repeated sprint training
SSG: Small-sided games
PRISMA: Preferred Reporting Items for Systematic Reviews and Meta-Analyses
p-BFR: Practical blood flow restriction
PEP: Pulse elimination pressure
AOP: Arterial occlusion pressure
$\dot{\mathrm{V}}{\mathrm{O}}_{2\mathrm{max}}$: Maximal oxygen uptake
${HR}_{\mathrm{max}}$: Maximal heart rate
MAP: Maximal aerobic power
CP: Critical power
PICO: Population, intervention, comparison, outcome
RSA: Repeated sprint ability
MPO: Mean power output
PPO: Peak power output
Sp ${\mathrm{O}}_{2}$: Pulse oxygen saturation
NIRS: Near infrared spectroscopy
ΔtHb: Changes in concentration for total haemoglobin
ΔHHb: Changes in concentration for deoxyhaemoglobin
ΔO_2_Hb: Changes in concentration for oxyhaemoglobin
TSI: Tissue saturation index
ΔTSIpfc: Changes in tissue saturation index on the pre-frontal cortex
Sm ${\mathrm{O}}_{2}$: Muscle oxygen saturation
TSI^bb^: Tissue saturation of biceps brachii
ΔTSI^bb^: Changes in tissue saturation of biceps brachii
bLa: Blood lactate
CK: Creatine kinase
p38MAPK: P38 mitogen-activated protein kinases
mRNA: Angiogenic messenger ribonucleic acid
PGC-1α: Peroxisome proliferator-activated receptor γ coactivator-1α
VEGF: Vascular endothelial growth factors
VEGF-2: Vascular endothelial growth factor receptors
HIF-1α: Hypoxia-inducible factor-1α
MVC: Maximal voluntary contraction
VAL: Voluntary action level
RMS/M-wave: Root mean square of muscle compound action potential
P10/P100: Ratio of resting stimulations at 10 Hz over stimulation at 100 Hz
ΔDb10: Change in force amplitude for paired electrical muscle stimulation at 10 Hz
Δ ${\mathrm{M}}_{\mathrm{max}}$: Change in amplitude of muscle compound action potential
PRV: Peak running velocity
$\dot{\mathrm{V}}{\mathrm{O}}_{2\mathrm{max}}$PRESS: Maximal oxygen uptake during incremental exercise test with BFR
IP-CE: Increasing BFR pressure with constant exercise intensity
CPp-IE: Constant partial BFR pressure with increasing exercise intensity
CPC-IE: Constant complete BFR pressure with increasing exercise intensity
IP-IE: Increasing BFR pressure with increasing exercise intensity
TT: Time trial
TTF: Time to fatigue
RE: Running economy
v $\dot{\mathrm{V}}{\mathrm{O}}_{2\mathrm{max}}$: Velocity at maximal oxygen uptake
RFD: Rate of force development
GH: Growth hormone
iPPO: Incremental knee extensor performance test
O_2_: Oxygen
iEMG: Integrated electromyography

## References

[1] Laursen P, Buchheit B. Science and Application of High-Intensity Interval Training: Human Kinetics; 2019.

[2] Scott BR, Loenneke JP, Slattery KM, Dascombe BJ (2015). Blood flow restricted exercise for athletes: a review of available evidence. J Sci Med Sport.

[3] Bennett H, Slattery F (2019). Effects of blood flow restriction training on aerobic capacity and performance: a systematic review. J Strength Cond Res.

[4] Silva JCG, Pereira Neto EA, Pfeiffer PAS, Neto GR, Rodrigues AS, Bemben MG (2019). Acute and chronic responses of aerobic exercise with blood flow restriction: a systematic review. Front Physiol.

[5] Formiga MF, Fay R, Hutchinson S, Locandro N, Ceballos A, Lesh A (2020). Effect of aerobic exercise training with and without blood flow restriction on aerobic capacity in healthy young adults: a systematic review with meta-analysis. Int J Sports Phys Ther.

[6] Ferguson RA, Mitchell EA, Taylor CW, Bishop DJ, Christiansen D. Blood-flow-restricted exercise: Strategies for enhancing muscle adaptation and performance in the endurance-trained athlete. Exp Physiol. 2021. 10.1113/EP089280 33486814

[7] Patterson SD, Hughes L, Warmington S, Burr J, Scott BR, Owens J (2019). Blood flow restriction exercise: considerations of methodology, application, and safety. Front Physiol.

[8] de Oliveira MF, Caputo F, Corvino RB, Denadai BS (2016). Short-term low-intensity blood flow restricted interval training improves both aerobic fitness and muscle strength. Scand J Med Sci Sports.

[9] Park S, Kim JK, Choi HM, Kim HG, Beekley MD, Nho H (2010). Increase in maximal oxygen uptake following 2-week walk training with blood flow occlusion in athletes. Eur J Appl Physiol.

[10] Paton CD, Addis SM, Taylor LA (2017). The effects of muscle blood flow restriction during running training on measures of aerobic capacity and run time to exhaustion. Eur J Appl Physiol.

[11] Christiansen D, Eibye K, Hostrup M, Bangsbo J (2020). Training with blood flow restriction increases femoral artery diameter and thigh oxygen delivery during knee-extensor exercise in recreationally trained men. J Physiol.

[12] Christiansen D, Eibye K, Hostrup M, Bangsbo J (2021). The effect of blood-flow-restricted interval training on lactate and H(+) dynamics during dynamic exercise in man. Acta Physiol (Oxf).

[13] Christiansen D, Eibye KH, Hostrup M, Bangsbo J (2019). Blood flow-restricted training enhances thigh glucose uptake during exercise and muscle antioxidant function in humans. Metabolism.

[14] Christiansen D, Eibye KH, Rasmussen V, Voldbye HM, Thomassen M, Nyberg M (2019). Cycling with blood flow restriction improves performance and muscle K(+) regulation and alters the effect of anti-oxidant infusion in humans. J Physiol.

[15] Amani-Shalamzari S, Rajabi S, Rajabi H, Gahreman DE, Paton C, Bayati M (2019). Effects of blood flow restriction and exercise intensity on aerobic, anaerobic, and muscle strength adaptations in physically active collegiate women. Front Physiol.

[16] Mitchell EA, Martin NRW, Turner MC, Taylor CW, Ferguson RA (2019). The combined effect of sprint interval training and postexercise blood flow restriction on critical power, capillary growth, and mitochondrial proteins in trained cyclists. J Appl Physiol..

[17] Elgammal M, Hassan I, Eltanahi N, Ibrahim H (2020). The effects of repeated sprint training with blood flow restriction on strength, anaerobic and aerobic performance in basketball. Int J Hum Movement Sports Sci.

[18] Keramidas ME, Kounalakis SN, Geladas ND (2012). The effect of interval training combined with thigh cuffs pressure on maximal and submaximal exercise performance. Clin Physiol Funct Imaging.

[19] Amani-Shalamzari S, Farhani F, Rajabi H, Abbasi A, Sarikhani A, Paton C (2019). Blood flow restriction during futsal training increases muscle activation and strength. Front Physiol.

[20] Amani-Shalamzari S, Sarikhani A, Paton C, Rajabi H, Bayati M, Nikolaidis PT (2020). Occlusion training during specific futsal training improves aspects of physiological and physical performance. J Sports Sci Med.

[21] Moher D, Liberati A, Tetzlaff J, Altman DG, Group P (2009). Preferred reporting items for systematic reviews and meta-analyses: the PRISMA statement. BMJ.

[22] Norton K, Norton L, Sadgrove D (2010). Position statement on physical activity and exercise intensity terminology. J Sci Med Sport.

[23] Hansen D, Bonne K, Alders T, Hermans A, Copermans K, Swinnen H (2019). Exercise training intensity determination in cardiovascular rehabilitation: Should the guidelines be reconsidered?. Eur J Prev Cardiol.

[24] Gibala MJ (2018). Intermittent exercise and insulin sensitivity in older individuals-It's a HIIT. Acta Physiol (Oxf).

[25] Huang X, Lin J, Demner-Fushman D. Evaluation of PICO as a knowledge representation for clinical questions. In: AMIA 2006 symposium proceedings. 2006:359. PMC1839740 17238363

[26] Peyrard A, Willis SJ, Place N, Millet GP, Borrani F, Rupp T (2019). Neuromuscular evaluation of arm-cycling repeated sprints under hypoxia and/or blood flow restriction. Eur J Appl Physiol.

[27] Taylor CW, Ingham SA, Ferguson RA (2016). Acute and chronic effect of sprint interval training combined with postexercise blood-flow restriction in trained individuals. Exp Physiol.

[28] Valenzuela PL, Sanchez-Martinez G, Torrontegi E, Vazquez-Carrion J, Gonzalez M, Montalvo Z, et al. Acute Responses to On-Court Repeated-Sprint Training Performed With Blood Flow Restriction Versus Systemic Hypoxia in Elite Badminton Athletes. Int J Sports Physiol Perform. 2019:1280–7. 10.1123/ijspp.2018-0878 30958054

[29] Willis SJ, Alvarez L, Borrani F, Millet GP (2018). Oxygenation time course and neuromuscular fatigue during repeated cycling sprints with bilateral blood flow restriction. Physiol Rep.

[30] Willis SJ, Borrani F, Millet GP (2019). Leg- vs arm-cycling repeated sprints with blood flow restriction and systemic hypoxia. Eur J Appl Physiol.

[31] Willis SJ, Peyrard A, Rupp T, Borrani F, Millet GP (2019). Vascular and oxygenation responses of local ischemia and systemic hypoxia during arm cycling repeated sprints. J Sci Med Sport.

[32] Behringer M, Behlau D, Montag JCK, McCourt ML, Mester J (2017). Low-intensity sprint training with blood flow restriction improves 100-m dash. J Strength Cond Res.

[33] Calbet JA, Holmberg HC, Rosdahl H, van Hall G, Jensen-Urstad M, Saltin B (2005). Why do arms extract less oxygen than legs during exercise?. Am J Physiol Regul Integr Comp Physiol.

[34] Calbet JA, Gonzalez-Alonso J, Helge JW, Sondergaard H, Munch-Andersen T, Saltin B (2015). Central and peripheral hemodynamics in exercising humans: leg vs arm exercise. Scand J Med Sci Sports.

[35] Wray DW, Uberoi A, Lawrenson L, Richardson RS (2005). Heterogeneous limb vascular responsiveness to shear stimuli during dynamic exercise in humans. J Appl Physiol..

[36] Delp MD, Laughlin MH (1998). Regulation of skeletal muscle perfusion during exercise. Acta Physiol Scand.

[37] Mortensen SP, Damsgaard R, Dawson EA, Secher NH, Gonzalez-Alonso J (2008). Restrictions in systemic and locomotor skeletal muscle perfusion, oxygen supply and VO2 during high-intensity whole-body exercise in humans. J Physiol.

[38] Curtelin D, Morales-Alamo D, Torres-Peralta R, Rasmussen P, Martin-Rincon M, Perez-Valera M (2018). Cerebral blood flow, frontal lobe oxygenation and intra-arterial blood pressure during sprint exercise in normoxia and severe acute hypoxia in humans. J Cereb Blood Flow Metab.

[39] Ganesan G, Cotter JA, Reuland W, Cerussi AE, Tromberg BJ, Galassetti P (2015). Effect of blood flow restriction on tissue oxygenation during knee extension. Med Sci Sports Exerc.

[40] Girard O, Mendez-Villanueva A, Bishop D (2011). Repeated-sprint ability - part I: factors contributing to fatigue. Sports Med.

[41] Pearcey GE, Bradbury-Squires DJ, Monks M, Philpott D, Power KE, Button DC (2016). Arm-cycling sprints induce neuromuscular fatigue of the elbow flexors and alter corticospinal excitability of the biceps brachii. Appl Physiol Nutr Metab.

[42] Amann M, Calbet JAL (2008). Convective oxygen transport and fatigue. J Appl Physiol.

[43] Amann M, Sidhu SK, Weavil JC, Mangum TS, Venturelli M (2015). Autonomic responses to exercise: Group III/IV muscle afferents and fatigue. Auton Neurosci.

[44] Blain GM, Mangum TS, Sidhu SK, Weavil JC, Hureau TJ, Jessop JE (2016). Group III/IV muscle afferents limit the intramuscular metabolic perturbation during whole body exercise in humans. J Physiol.

[45] Bishop D, Edge J, Goodman C (2004). Muscle buffer capacity and aerobic fitness are associated with repeated-sprint ability in women. Eur J Appl Physiol.

[46] Thomas C, Perrey S, Lambert K, Hugon G, Mornet D, Mercier J (2005). Monocarboxylate transporters, blood lactate removal after supramaximal exercise, and fatigue indexes in humans. J Appl Physiol..

[47] Glaister M (2005). Multiple sprint work : physiological responses, mechanisms of fatigue and the influence of aerobic fitness. Sports Med.

[48] Wernbom M, Paulsen G, Nilsen TS, Hisdal J, Raastad T (2012). Contractile function and sarcolemmal permeability after acute low-load resistance exercise with blood flow restriction. Eur J Appl Physiol.

[49] Umbel JD, Hoffman RL, Dearth DJ, Chleboun GS, Manini TM, Clark BC (2009). Delayed-onset muscle soreness induced by low-load blood flow-restricted exercise. Eur J Appl Physiol.

[50] McKay AKA, Stellingwerff T, Smith ES, Martin DT, Mujika I, Goosey-Tolfrey VL (2022). Defining training and performance Caliber: a participant classification framework. Int J Sports Physiol Perform.

[51] Stone M, Plisk S, Collins D (2002). Training principles: evaluation of modes and methods of resistance training–a coaching perspective. Sports Biomech.

[52] Gist NH, Fedewa MV, Dishman RK, Cureton KJ (2014). Sprint interval training effects on aerobic capacity: a systematic review and meta-analysis. Sports Med.

[53] Macpherson RE, Hazell TJ, Olver TD, Paterson DH, Lemon PW (2011). Run sprint interval training improves aerobic performance but not maximal cardiac output. Med Sci Sports Exerc.

[54] Thomas K, Goodall S, Stone M, Howatson G, St Clair Gibson A, Ansley L (2015). Central and peripheral fatigue in male cyclists after 4-, 20-, and 40-km time trials. Med Sci Sports Exerc..

[55] Abe T, Satoshi F, Toshiaki NSM, Hayao O, Riki O (2010). Effects of low-intensity cycle training with BFR on muscle volume and VO_2__max_, in young men. J Sports Sci Med..

[56] Scott BR, Slattery KM, Sculley DV, Dascombe BJ (2014). Hypoxia and resistance exercise: a comparison of localized and systemic methods. Sports Med.

[57] Abe T, Kearns CF, Sato Y (2006). Muscle size and strength are increased following walk training with restricted venous blood flow from the leg muscle Kaatsu-walk training. J Appl Physiol..

[58] Brandner CR, Warmington SA, Kidgell DJ (2015). Corticomotor excitability is increased following an acute bout of blood flow restriction resistance exercise. Front Hum Neurosci.

[59] Loenneke JP, Kim D, Fahs CA, Thiebaud RS, Abe T, Larson RD (2015). Effects of exercise with and without different degrees of blood flow restriction on torque and muscle activation. Muscle Nerve.

[60] Yasuda T, Fukumura K, Fukuda T, Iida H, Imuta H, Sato Y (2014). Effects of low-intensity, elastic band resistance exercise combined with blood flow restriction on muscle activation. Scand J Med Sci Sports.

[61] Bailey SJ, Wilkerson DP, Dimenna FJ, Jones AM (2009). Influence of repeated sprint training on pulmonary O2 uptake and muscle deoxygenation kinetics in humans. J Appl Physiol..

[62] Yasuda T, Loenneke JP, Ogasawara R, Abe T (2013). Influence of continuous or intermittent blood flow restriction on muscle activation during low-intensity multiple sets of resistance exercise. Acta Physiol Hung.

[63] Fitschen PJ, Kistler BM, Jeong JH, Chung HR, Wu PT, Walsh MJ (2014). Perceptual effects and efficacy of intermittent or continuous blood flow restriction resistance training. Clin Physiol Funct Imaging.

[64] Olney N, Wertz T, LaPorta Z, Mora A, Serbas J, Astorino TA (2018). Comparison of acute physiological and psychological responses between moderate-intensity continuous exercise and three regimes of high-intensity interval training. J Strength Cond Res.

[65] Rosenblat MA, Perrotta AS, Thomas SG (2020). Effect of high-intensity interval training versus sprint interval training on time-trial performance: a systematic review and meta-analysis. Sports Med.

[66] Wood KM, Olive B, LaValle K, Thompson H, Greer K, Astorino TA (2016). Dissimilar physiological and perceptual responses between sprint interval training and high-intensity interval training. J Strength Cond Res.

[67] Millet GP, Vleck VE, Bentley DJ (2009). Physiological differences between cycling and running: lessons from triathletes. Sports Med.

